# Effect of Relaxations on the Conductivity of La_1/2+1/2*x*_Li_1/2–1/2*x*_Ti_1–*x*_Al*_x_*O_3_ Fast Ion Conductors

**DOI:** 10.1021/acs.chemmater.2c00459

**Published:** 2022-06-06

**Authors:** Keti Vezzù, Ester García-González, Gioele Pagot, Esteban Urones-Garrote, Maria Eugenia Sotomayor, Alejandro Varez, Vito Di Noto

**Affiliations:** † Section of Chemistry for the Technology (ChemTech), Department of Industrial Engineering, University of Padova, Via Marzolo 9, I-35131 Padova (PD), Italy; ‡ Departamento de Química Inorgánica. Facultad de Ciencias Químicas, Universidad Complutense, Madrid 28040, Spain; § Centro Studi di Economia e Tecnica dell’Energia Giorgio Levi Cases, Via Marzolo 9, I-35131 Padova (PD), Italy; ∥ Centro Nacional de Microscopia electrónica, Facultad de Ciencias Químicas, Universidad Complutense, Madrid 28040, Spain; ⊥ Materials Science and Engineering Department, University Carlos III of Madrid, Av. de la Universidad 30, Leganés, Madrid E-28911, Spain

## Abstract

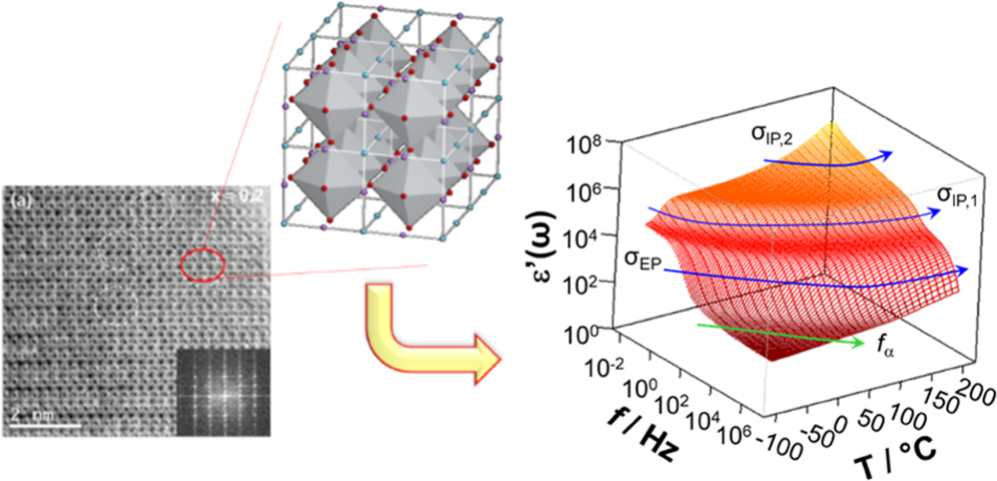

Perovskite-type solid-state electrolytes, Li_3*x*_La_2/3–*x*_TiO_3_ (LLTO), are considered among the most promising candidates for the development of all-solid-state batteries based on lithium metal. Their high bulk ionic conductivity can be modulated by substituting part of the atoms hosted in the A- or B-site of the LLTO structure. In this work, we investigate the crystal structure and the long-range charge migration processes characterizing a family of perovskites with the general formula La_1/2+1/2*x*_Li_1/2–1/2*x*_Ti_1–*x*_Al*_x_*O_3_ (0 ≤ *x* ≤ 0.6), in which the charge balance and the nominal A-site vacancies (*n*_A_ = 0) are preserved. X-ray diffraction (XRD) and high-resolution transmission electron microscopy (HRTEM) investigations reveal the presence of a very complex nanostructure constituted by a mixture of two different ordered nanoregions of tetragonal *P*4/*mmm* and rhombohedral *R*3̅*c* symmetries. Broadband electrical spectroscopy studies confirm the presence of different crystalline domains and demonstrate that the structural fluctuations of the BO_6_ octahedra require to be intra- and intercell coupled, to enable the long-range diffusion of the lithium cation, in a similar way to the segmental mode that takes place in polymer-ion conductors. These hypotheses are corroborated by density functional theory (DFT) calculations and molecular dynamic simulations.

## Introduction

The all-solid-state batteries (ASSBs) using solid electrolytes are excellent candidates for next-generation commercial devices for energy storage.^1−12^ Among these, the perovskite-type family of Li-ion-conducting oxides Li_3*x*_La_2/3–*x*_TiO_3_ (LLTO) is one of the most studied,^13^ and it is shown as the most promising option for solid electrolytes because of its high bulk conductivity (10^–3^ S·cm^–1^),^14^ negligible electronic conductivity,^15^ high stability, wide electrochemical window (larger than 4 V),^16^ and easy preparation.^17^ However, there are still many challenges to be solved: (i) the high impedance associated with grain boundaries, which reduce the overall lithium ionic conductivity below 10^–5^ S·cm^–1^ at 298 K; and (ii) the instability of LLTO in direct contact with metal Li^18,19^ or graphite electrodes owing to the ease of reduction of Ti^4+^ to Ti^3+^, thus leading to an increase in electronic conductivity, which yields undesired short-circuits.^20−22^

To understand the high ionic conductivity of the Li_3*x*_La_2/3–*x*_TiO_3_ materials, several chemical substitutions have been performed on this material, both in A (La, Li) and in B (Ti) sites.^17^ However, a clear understanding of the relationship existing between the structure and/or the microstructure and the high ionic conductivity of these systems is still missing. As a general fact, Li-ion conductivity is highly dependent on the crystal structure, composition (*e.g*., charge carrier and vacancy concentration), and structural distortions. Depending on the composition and heat treatment, the A-site cations and vacancies can be randomly distributed or ordered along the [001] direction to form alternately stacked La-rich (La1) and La-poor (La2) layers. This fact determines the dimensionality of the Li trajectories. In the so-called ordered samples, Li atoms are predominantly confined within the La-poor layer^23^ and the La-rich layer acts as a barrier to Li^+^ migration along the *c*-axis, resulting in a two-dimensional (2D) mobility. In disordered samples (*i.e*., where La and vacancies are randomly distributed along one single A-site), the random location of Li atoms in sites facilitates the three-dimensional (3D) network fluctuations of perovskites.^23^ The unusual position of the Li ion in these perovskites is a key factor to explain the very high ionic conductivity. Diverse studies have shown that Li atoms are located in the oxygen square windows between adjacent A-sites,^23−25^ thus resulting in a number of A vacant sites, which is considerably higher than the nominal amount. This finding brings the concept of “effective vacancy” (*n*_t_) instead of “nominal vacancy” (□_A_) as the most relevant parameter for controlling Li diffusion, which is defined as *n*_t_ = [Li]+ □_A_. Under this perspective, it has been possible to easily interpret the evolution of conductivity with the number of effective vacancies, in different solid solutions, observing the phenomenon of Li movement blocking by eliminating A-site vacancies, following a clear percolation mechanism.^25−27^

In addition to high bulk ionic conductivity, low grain boundary resistance is necessary in a good candidate as a solid electrolyte in an ASSB. Unfortunately, in these perovskites, the total conductivity decreases from 1 to 2 orders of magnitude because of the grain boundaries (σ_T_ ∼ 10^–4^–10^–5^ S·cm^–1^). Through high-resolution transmission electron microscopy (HRTEM), and from the beginning of its discovery, the presence of complex microstructures in LLTO systems was suggested, and it is well accepted that crystals are often formed by structural domains with different crystal orientations and periodicities.^28,29^ Experimental evidence of the relationship between conductivity and microstructures (domain size and composition of domain boundaries) has been provided,^30,31^ showing that Li mobility in LTO is strongly affected by 90° domain boundaries and that dramatic structural and chemical deviations can be observed at most grain boundaries.

In the present work, we have focused our attention on a particular case of aliovalent substitution on the B-site of the LLTO perovskite, La_1/2+1/2*x*_Li_1/2–1/2*x*_Ti_1–*x*_Al*_x_*O_3_ (0 ≤ *x* ≤ 0.6), with the aim to analyze the influence of the micro- and nanostructure on the long-range charge migration phenomena. Along this series, (1/2Li^+^ + Ti^4+^) cations are substituted by (1/2La^3+^ + Al^3+^), preserving both the charge balance and the nominal A-site vacancies (*n*_A_ = 0). A recent study of this solid solution has shown an upward deviation from the linear ideal Vegard’s law, which was tentatively associated with a partial order or segregation of the distribution of the nonisovalent cations involved in the solid solution.^27^ The presence of this order/segregation phenomenon is expected to influence the conductivity of these materials. The structural features have been studied in detail by combining X-ray diffraction (XRD), HRTEM, and associated techniques. The relaxation events of these materials have been investigated by means of broadband electric spectroscopy, which is a powerful technique for unraveling the complex dynamics of a material. The aim here is to investigate the influence of the nanostructural features of the proposed polycrystalline ceramic materials on their electric response. To better elucidate the existing relationships among structural features, conductivity, and the relaxation phenomena characterizing the electric response of these perovskites, density functional theory (DFT) and dynamic molecular modeling simulation studies have been carried out.

## Experimental Section

### Synthesis

Ceramic samples of nominal composition La_1/2+1/2*x*_Li_1/2–1/2*x*_Ti_1–*x*_Al*_x_*O_3_ (0.1 ≤ *x* ≤ 0.6) were synthesized from stoichiometric amounts of La_2_O_3_ (99.99% Aldrich), Li_2_CO_3_ (99.9%, Aldrich), TiO_2_ (99.99%), and Al(NO_3_)_3_·9H_2_O (>98% Aldrich) by a solid-state reaction as previously described.^32^ These reagents were ground together in an agate mortar and heated at 800 °C for 12 h in highly dense alumina crucibles (La_2_O_3_ was previously dried at 700 °C for 4 h for decarbonation). The reground powders were isostatically cold-pressed at 200 MPa and heated at 1100 °C for 24 h. Finally, they were uniaxial-pressed and heated again for 6 h at a temperature range of 1300–1400 °C depending on their composition and furnace-cooled to room temperature. To avoid lithium losses, the heating rate in the thermal treatment was 1 °C·min^–1^.

### Structure and Chemical Composition

The chemical composition of the bulk samples was determined by inductively coupled plasma atomic emission spectroscopy (ICP SPECTRO Arcos with EndOnPlasma torch). The emission lines are as follows: 394.401, 333.749, 670.780, and 336.121 nm for Al, La, Li, and Ti, respectively. Room-temperature powder X-ray diffraction (XRD) patterns were obtained by a Panalytical X’PERT PRO ALPHA 1 diffractometer with a Ge(111) primary beam monochromator prealigned for Cu Kα1 radiation (λ = 1.540598 Å) with an X’Celerator fast detector. Data were collected in the 2⊖ range of 5–110° with an effective step time of 800 s and a step size of 0.017 (°2Θ). The refinement of the crystal structure was performed by the Rietveld method using the FULLPROF package program.^33^ The powder density of synthesized perovskites was determined by a helium gas pycnometer (Micromeritics Accupyc 1330 pycnometer).

### Electron Microscopy Studies

Samples for transmission electron microscopy (TEM) were ultrasonically dispersed in n-butanol and transferred to carbon-coated copper grids. Selected area electron diffraction (SAED) and high-resolution transmission electron microscopy (HRTEM) were performed on a JEOL JEM300F electron microscope working at 300 kV (point resolution of 0.17 nm). Crystal-by-crystal chemical microanalysis was performed by energy-dispersive X-ray spectroscopy (XEDS) in the same microscope equipped with an ISIS 300 X-ray microanalysis system (Oxford Instruments) with a detector model LINK “Pentafet” (resolution 135 eV). High-angle annular dark-field (HAADF) and annular bright-field (ABF) scanning TEM (STEM) studies were performed on an ARM200cF microscope, fitted with a condenser lens aberration corrector (point resolution in the STEM mode of 0.08 nm). HAADF images were acquired with an inner acceptance angle of 90 mrad, and ABF ones with a collection angle of 11 mrad. The same ARM200cF microscope was used for electron energy-loss spectroscopy (EELS) experiments since it is fitted with a GIF Quantum-ER spectrometer. EELS mapping was performed with a collection semiangle β ∼ 30 mrad, 1 eV per channel dispersion, and a collection time for each spectrum of 0.1 s. La-M4,5, Ti-L2,3, and Al-K edge signals were chosen for mapping.

### Broadband Electric Spectroscopy Studies

Electric response was measured by broadband electric spectroscopy (BES) in the frequency range from 30 mHz to 10^7^ Hz using a Novocontrol Alpha-A analyzer over the temperature range from −100 to 150 °C. The temperature was controlled using a homemade cryostat operating with a N_2_ gas jet heating and cooling system. The temperature was measured with an accuracy greater than ±0.2 °C. The measurements were carried out on disks with a diameter of 12 mm and a thickness of 1 mm. These latter were prepared by a uniaxial hot pressing process at 1200 °C for 6 h. Gold electrodes (Dupont QG150 Au paste) were painted onto polished surfaces of the sintered ceramic discs and subsequently heated at 850 °C for 1 h. The cell was closed in a glovebox filled with argon and maintained under nitrogen during the measurements. All of the samples were dried at 60 °C under a vacuum for at least 48 h. The geometrical cell constant was determined from the electrode–electrolyte contact surface. The distance between electrodes was determined using a micrometer. Corrections for thermal expansion of the cell were not adopted.

### Computational Studies

The atomistic DFT and simulation computational methods used in this study were carried out using Castep and Forcite programs, respectively, operating within the Materials Studio 2016 package.^34^ Geometry optimization of structural models is performed by adopting a simulation box with periodic boundary conditions consisting of 6 × 3 × 3 unit cells (in the box are included approximately 500 atoms). GGA and PBESOL are used as pseudopotential functionals and OFTG ultrasoft as the pseudopotential set, respectively. The molecular dynamic (MD) simulations were carried out using the Forcite module by adopting a simulation box of 6 × 5 × 5 unit cells. The system is first equilibrated under a constant pressure of 1 atm and then studied in the NVT ensemble (Nose–Hoover thermostat) at 298 K for 10 ps (step of 1 fs).

## Results and Discussion

### Structural-Microstructural Characterization

The powder XRD patterns of the solid solution La_1/2+1/2*x*_Li_1/2–1/2*x*_Ti_1–*x*_Al*_x_*O_3_ (0.1 ≤ *x* ≤ 0.6) were analyzed in a previous paper.^32^ It was pointed out the existence of a certain extra order or phase segregation at the nanostructural level. To study this effect in depth, we have focused our attention on three compositions of the series, *i.e*., *x* = 0.2, 0.4, and 0.6.

**Figure 1:**
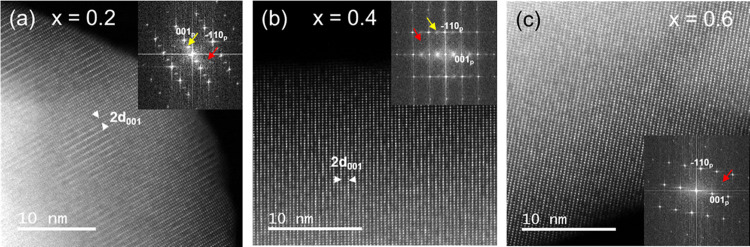
HAADF images of crystals of the *x* = 0.2 (a), *x* = 0.4 (b), and *x* = 0.6 (c) compositions in the [110]_p_ zone axis. The corresponding Fourier transforms have been included as insets. Yellow arrows indicate diffraction maxima at 1/2 {001}_p_ and red arrows those at 1/2 {111}_p_.

**Figure 2:**
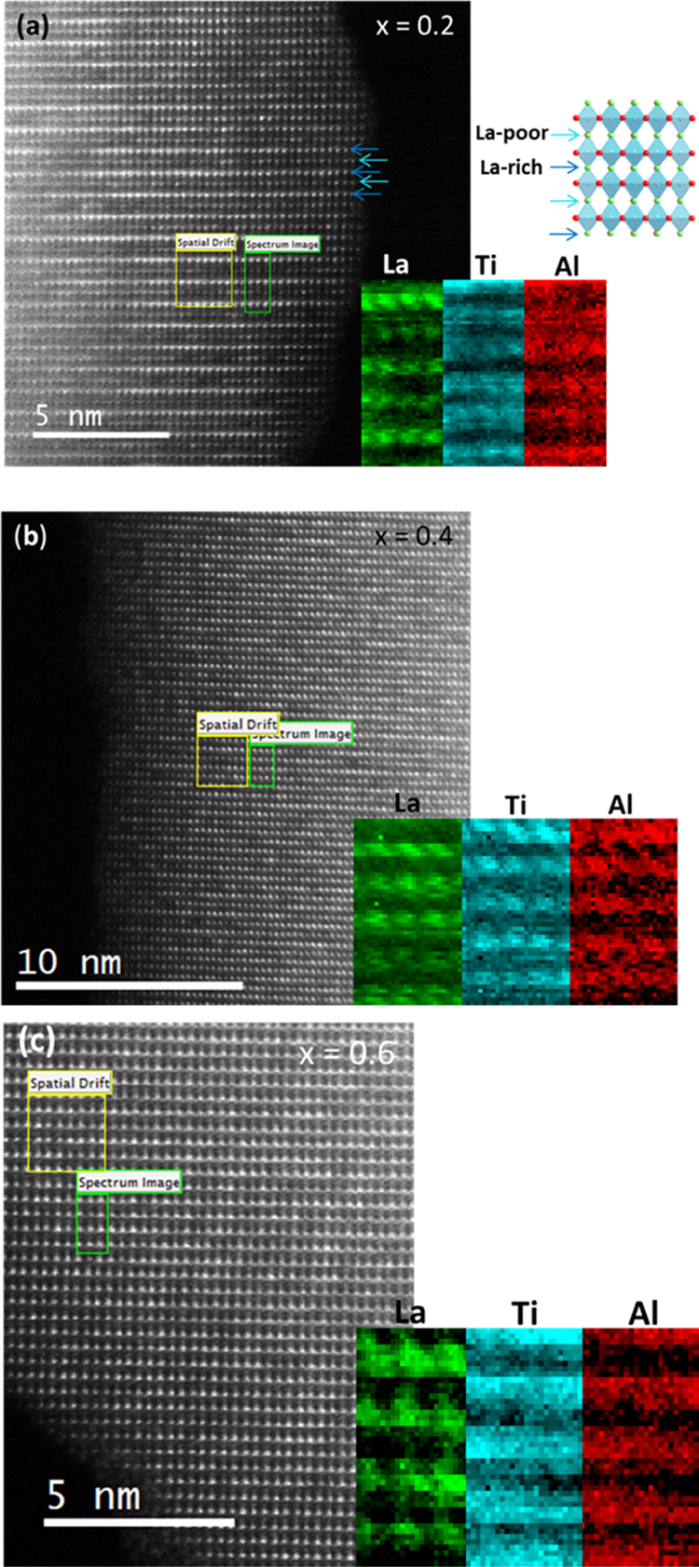
HAADF images of crystals of the *x* = 0.2 (a), *x* = 0.4 (b), and *x* = 0.6 (c) compositions. Atomic-resolution elemental mapping from the La-M4,5, Ti-L2,3, and Al-K edge EELS signals has been recorded from the selected areas marked with green squares.

**Figure 3:**
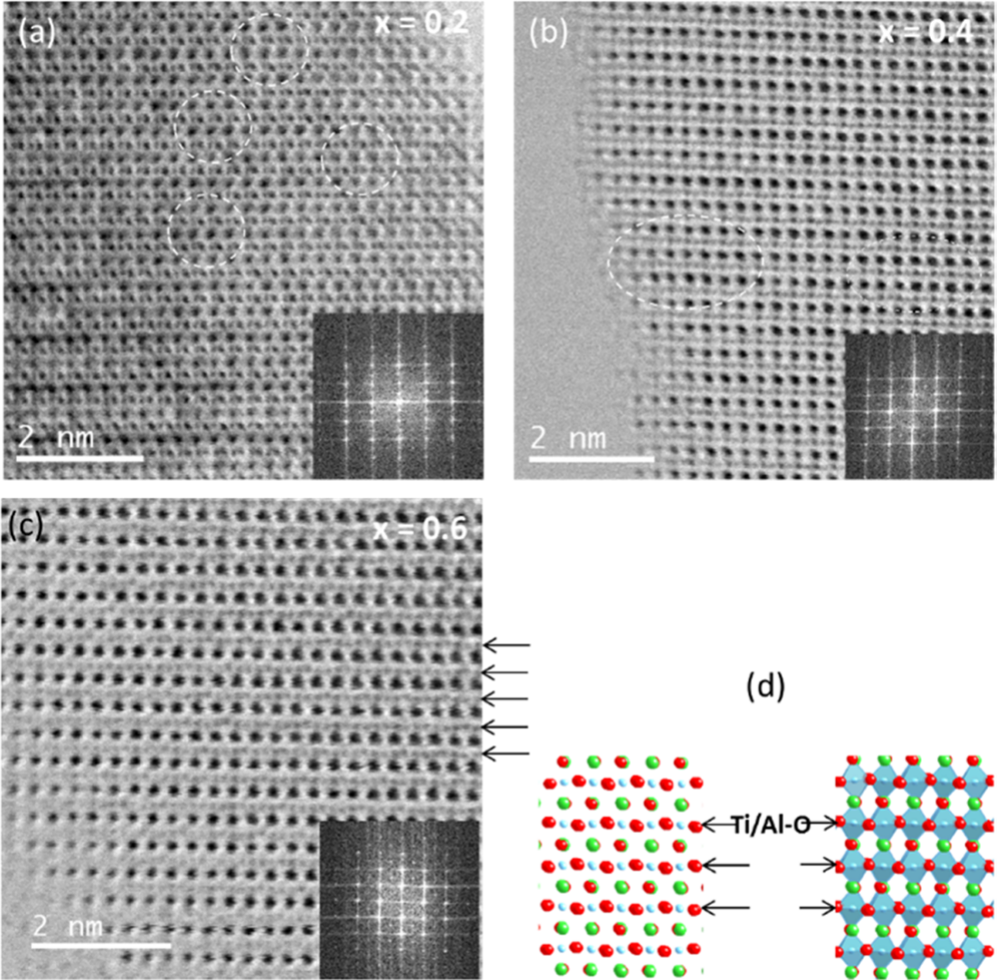
ABF images of crystals of the *x* = 0.2 (a), *x* = 0.4 (b), and *x* = 0.6 (c) compositions, in the [110] projection. Encircled areas have been selected to show an evident Ti/Al–O misalignment. Corresponding projected structure model where Ti/Al–O layers have been labeled (d).

The general electric response of the materials as well as their very high dielectric constant at low frequencies prompted the necessity of performing a detailed microstructural study by means of transmission electron microscopy. From the first observations, samples appear to have a very complex nanostructure. Figure S1 in the Supporting Information shows the high-resolution electron micrographs of crystals corresponding to *x* = 0.2 (a), 0.4 (b), and 0.6 (c) compositions in the [110]_p_ zone axis (the p subindex refers to the basic perovskite cell). Images show regions with double contrast with periodicity 2*d*_001p_, which tend to disappear for increasing concentrations of aluminum. The corresponding Fourier transform displayed as the inset exhibits features that account for the observed contrasts. A double periodicity along the [001] reciprocal direction is visible, and the intensity of the corresponding diffraction maxima diminishes when increasing the aluminum content. This is compatible with a doubled tetragonal perovskite (*a*_p_ × *a*_p_ × 2*a*_p_) with the *P*4/*mmm* space group, which considered layered cation ordering of the A-sites along the *c*-axis in (AA′)BO_3_ perovskites.^35^ For the sake of clarity, the structural features of different space groups of perovskites are shown in Figure S2. This symmetry has been previously observed, for a certain composition range, in LLTO and in different systems derived from chemical substitution.^32^ In addition, there are maxima doubling [111]_p_ and equivalent reciprocal directions that can be observed along the series, which do not match the assumed tetragonal symmetry. However, it is difficult to distinguish any other extra feature contrast in the micrographs that accounts for the new diffraction maxima. Samples were further investigated by simultaneous HAADF and ABF-STEM imaging. Image contrast under HAADF conditions is roughly proportional to the atomic number of the species. Chemical sensitivity, however, is insignificant for light atoms such as lithium and oxygen. On the contrary, under ABF imaging conditions, the contrast has a low scaling rate with *Z*, thus being a powerful technique for simultaneous imaging of light and heavy elements. Figure 1a–c corresponds to the HAADF images of crystals with *x* = 0.2, 0.4, and 0.6 compositions, respectively. The images demonstrate that the double periodicity 2*d*_001p_ observed along the series is in fact chemical contrast. Layers of bright dots corresponding to columns of La atoms alternate in an ordered way and can be assigned to the La-rich and La-poor layers of the structural model, provided their difference in brightness. They account for the double periodicity along the *c*-axis and are easily observed in the crystals with *x* = 0.2 and 0.4 compositions but are practically indistinguishable for *x* = 0.6. Atomic-resolution elemental mapping from the La-M4,5, Ti-L2,3, and Al-K edge EELS signals has been recorded from the selected areas marked with green squares in Figure 2a–c. From the analysis performed for the different compositions, it is clear that alternating La-rich/La-poor positions occur and that Ti and Al atoms are randomly distributed in the same crystallographic positions. There are no other distinguishable features in the HAADF images that account for the extra diffraction maxima 1/2 {111}_p_ in spite of the fact that they are visible in the corresponding FFT (insets in Figures S1 and 2). Maxima of the type 1/2 {111}_p_ are consistent with a rhombohedral distorted *R*3̅*c* cell, where octahedral tilting about [111]_p_ gives rise to this extra diffraction feature (Figure S2 shows the space groups of perovskites). This rhombohedral cell has been reported for quenched samples in Li-rich LLTO compounds as well as for La_0.5_Li_0.5_TiO_3_.^23,24^ Therefore, this type of distortion has its origin in the oxygen sublattice, and oxygen and lithium atoms do not contribute to the image contrast under HAADF imaging conditions. Figure 3a–c shows the ABF images of crystals with *x* = 0.2, 0.4, and 0.6 compositions, respectively, in the [110] projection. Projected columns of oxygen atoms are clearly resolved under these imaging conditions. Evident misalignment is observed along the Ti(Al)-O layers in the *x* = 0.6 material, where oxygen atoms are arranged up and down alternately around the titanium atoms, thus giving rise to octahedra rotation. This is restricted to specific areas of the crystal for composition 0.4, and the regions are even smaller when we look at the image corresponding to the material *x* = 0.2 (see the encircled areas in Figure 3a,b). To make the observation easier, the corresponding projected structure model is shown in Figure 3d, where Ti/Al–O layers have been labeled.

All of the above results led us to the conclusion that crystals of the different compositions in the La_1/2+1/2*x*_Li_1/2–1/2*x*_Ti_1-*x*_Al*_x_*O_3_ series are formed by the disordered intergrowth of structural domains of the tetragonal and rhombohedral crystal phases. From our observations, their relative size and amount change across the series, with the tetragonal cell domains diminishing on increasing the aluminum content: ∼10–12 nm for *x* = 0.2 to ∼4–5 nm for *x* = 0.4, and they can hardly be distinguished (1–2 unit cells) from the image contrast with *x* = 0.6. At the same time, regions of the rhombohedral crystal phase increase their relative size as the Al content increases and can be considered nearly the only constituent of crystals with *x* = 0.6 composition. In addition, the characterization performed has shown that there is neither chemical change in composition nor chemical segregation associated with the different structural domains. At this point, it is important to note that a similar microstructural behavior has been found in the La_0.5–*x*_Li_0.5–*x*_Sr_2*x*_TiO_3_ series, where the same crystal structures are found to grow endotaxially related at the nanoscale, thus constituting the crystals.^36^ Competition between these two distortive processes is well known in perovskites with different A cations having important size mismatch, with one process prevailing over the other depending on the nature and distribution of A cations, which affects the lowest energy structure. In these two series, the structure is not fully stable with respect to either the tilting distortions or the layered distortive phenomenon, and systems display the capability of simultaneously minimizing the competing interaction energy between the two processes. Thus, the final structure is a mixture of two different ordered regions in the absence of long-range periodic correlations.

From the above information, Rietveld analysis of the X-ray diffraction data was performed using Fullprof software. A mixture of the two observed crystal phases was considered. The structural models used in the refinement are indicated in Table S1 of the Supporting Information.^24,37^ Due to their low scattering factor, Li atoms were not considered in the refinement. In the case of the tetragonal phase (space group *P*4/*mmm*), La atoms were located on two nonequivalent positions to account for the double periodicity observed and assigned to the alternation of La-rich and La-poor layers along the [001] direction. In the case of the rhombohedral phase (space group *R*3̅*c*), La atoms are placed in a unique site, at 6*a* (0,0,1/4). In both structural models, Ti and Al were randomly distributed in the same atomic position.

**Figure 4:**
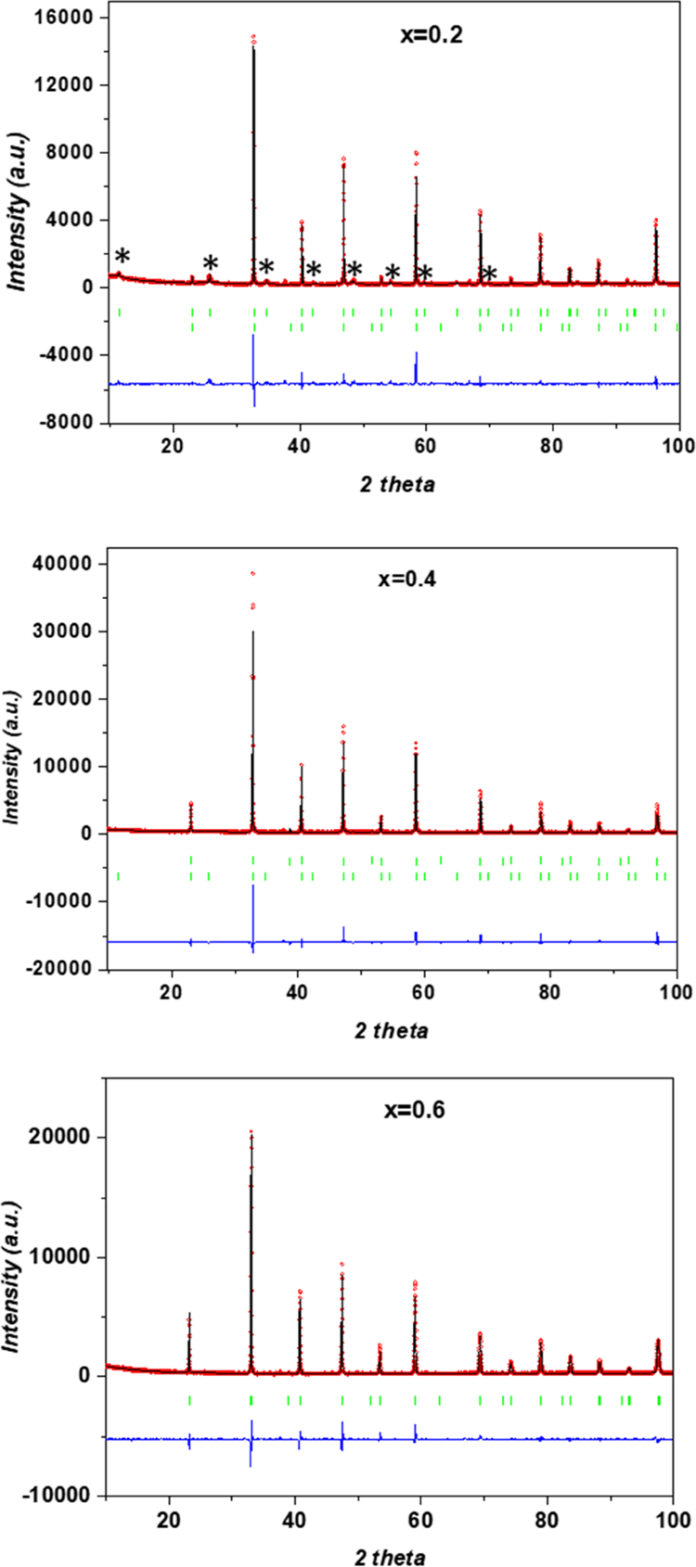
Powder XRD profiles of selected La_1/2+1/2*x*_Li_1/2–1/2*x*_Ti_1–*x*_Al*_x_*O_3_ perovskite samples. Fitted curves are obtained by Rietveld analysis considering a mixture of rhombohedral *R*3̅*c* (N° 167) and tetragonal *P*4/*mmm* (N° 123) space groups.

Figure 4 shows the XRD patterns of the three samples analyzed, as well as the refinement results. The summary of structural parameters and agreement factors obtained in this analysis is given in Table 1. The interatomic distances as deduced from the refinement are displayed in Table 2. For the *x* = 0.2 sample, the characteristic peaks related to the doubled cell (*a*_p_ × *a*_p_ × 2*a*_p_), labeled with asterisks, are clearly observed. These peaks are broad in comparison with those of the pseudo-cubic perovskite (*a*_p_ × *a*_p_ × *a*_p_) sublattice, which is associated with nanodomain formation. The asymmetric distribution of lanthanum between La1 and La2 sites provokes a coordinated displacement of titanium atoms from the octahedra center toward La2, where vacancies are preferably located (see Table 2). Thus, different B–O distances appear along the *c*-axis, 1.824 and 2.045 Å, and four distances close to 1.936 Å in the *ab* plane. In La-rich planes, the mean La1–O distance (⟨La1–O⟩ = 2.755 Å) is longer than that in La-poor planes (⟨La2–O⟩ = 2.728 Å). Octahedra distortion also causes distortion of the oxygen square windows that connect the contiguous A-site of the perovskite. Diagonal distances along *a* and *b* axes are longer than along the *c*-axis, indicating that diffusion should be favored in the *ab* plane (2D movement) The percentage of the tetragonal phase is 63.7% as calculated from the refinement.

**Table 1:** Structural Parameters Deduced from X-ray Powder Diffraction Data of La_1/2+1/2*x*_Li_1/2–1/2*x*_Ti_1–*x*_Al*_x_*O_3_ Perovskites^a^^,^^b^

S.G.	*x*	0.2 (2Ph)	0.4 (2Ph)	0.6 (1Ph)
*R*3̅*c*	*a* (Å)	5.4729(2)	5.4479 (3)	5.4214(1)
	*c* (Å)	13.408(1)	13.343(1)	13.2534(1)
	O (*x*)	0.5229(5)	0.4719(1)	0.4671(1)
	La (occ)	0.600	0.701	0.801
	Al (occ)	0.188	0.403	0.454
	Ti (occ)	0.812	0.597	0.546
	O (occ)	3.000	3.000	3.000
	*R*_B_	5.92	5.41	6.56
	*R*_F_	6.49	4.34	3.29
	%phase	36.3(3)	96.2(3)	100
*P*4/*mmm*	*a* (Å)	3.8700(1)	3.8516(2)	
	*c* (Å)	7.7364(5)	7.705(3)	
	*c*/2*a*	0.9995	1.0001	
	Ti (*z*)	0.264(1)	0.261(5)	
	O3 (*z*)	0.255(2)	0.249(8)	
	La1 (occ)	0.369	0.573	
	La2 (occ)	0.231	0.514	
	Al (occ)	0.331	0.282	
	Ti (occ)	0.834	0.718	
	O1 (occ)	0.500	0.5	
	O2 (occ)	0.5	0.5	
	O3 (occ)	2	2	
	*R*_B_	10.6	7.47	
	*R*_F_	9.38	7.47	
	%phase	63.7(2)	3.8(1)	
	*R*_p_	7.21	6.06	7.18
	*R*_wp_	9.76	8.74	10.7
	χ^2^	3.66	3.80	4.59

a All of the patterns were refined considering a mixture (2Ph) of rhombohedral *R*3̅*c* (N° 167) and tetragonal *P*4/*mmm* (N° 123) space groups. In the cases of *x* = 0.6 and 0.4, they have also been refined considering the rhombohedral single phase (1Ph).

b *R*_p_, *R*_wp_, χ^2^, and *R*_B_ are conventional agreement factors given by the refinement program.

**Table 2:** M–O and O–O Distances Deduced from X-ray Powder Diffraction Data of La_1/2+1/2*x*_Li_1/2–1/2*x*_Ti_1–*x*_Al*_x_*O_3_ Perovskites

S.G.	*x*	0.2 (2Ph)	0.4 (2Ph)	0.6 (1Ph)
*R*3̅*c*				
	6 × d_Ti-O_(Å)	1.9391(2)	1.932(1)	1.92378(12)
	3 × d_La-O_(Å)	2.611(1)	2.571(8)	2.53229(16)
	6 × d_La-O_(Å)	2.740(1)	2.728(1)	2.71301(9)
	3 × d_La-O_(Å)	2.862(1)	2.876(8)	2.88913(8)
d(O–O)_l_^a^	2 × d_O1-O1_(Å)	4.051(3)	4.074(2)	4.09004(17)
d(O–O)_s_^a^	2 × d_O1-O1_(Å)	3.697(3)	3.643(1)	3.58845(15)
d_∥_^O–O^	4 × d_O1-O1_(Å)	2.745(2)	2.737(1)	2.72827(9)
d_⊥_^O–O^	4 × d_O1-O1_(Å)	2.740(2)	2.728(1)	2.71301(12)
*P*4/*mmm*				
	4 × d_La1-O1_(Å)	2.73657(7)	2.7234(9)	
	8 × d_La1-O3_(Å)	2.7640(1)	2.72(4)	
	4 × d_La2-O2_(Å)	2.73657(7)	2.7235(9)	
	8 × d_La2-O3_(Å)	2.7083(1)	2.73(4)	
	1 × d_Ti-O1_(Å)	2.0449(1)	2.01(4)	
	1 × d_Ti-O2_(Å)	1.8236(1)	1.84(4)	
	4 × d_Ti-O3_(Å)	1.93636(6)	1.928(3)	
d_∥_^O–O^ (La1)	4 × d_O1-O1_(Å)	3.8701(1)	3.8516(2)	
d_∥_^O–O^ (La2)	4 × d_O2-O2_(Å)	3.8701(1)	3.8516(2)	
d_⊥_^O–O^ (La1)	1 × d_O3-O3_(Å)	3.9472(3)	3.87(9)	
d_⊥_^O–O^ (La2)	1 × d_O3-O3_(Å)	3.7897(2)	3.84(9)	

a d(O–O)_s_ and d(O–O)_l_ correspond to the two nonequivalent short and long sides of the square window.

In the cases of *x* = 0.4 and 0.6 samples, the broad superstructure peaks cannot be distinguished. From the results obtained in the microstructural characterization, in which the presence of BO_6_ octahedral tilting is evidenced, the rhombohedral *R*3̅*c* symmetry with a √2*a*_p_ × √2*a*_p_ × 2√3*a*_p_ unit cell was considered in the refinement (see Table SI1). The regular octahedral network gives rise to a single B–O distance, but tilting produces a distortion of the oxygen square windows that connect contiguous A-sites, and two different O–O distances (d(O–O)_l_ and d(O–O)_s_) are found. For *x* = 0.4, electron microscopy provides direct evidence of the presence of domains of the tetragonal phase; however, the corresponding X-ray diffraction maxima cannot be distinguished, proving that the average domain size is smaller than the coherence length in this case. To reconcile both results, structural refinement was performed by considering the two crystal phases, and the percentage of the tetragonal phase was ∼4%. For *x* = 0.6, however, the microstructural analysis revealed that the presence of the tetragonal phase was negligible, thus allowing a reliable matching of the experimental data to the rhombohedral phase.

### Electric Response

**Figure 5:**
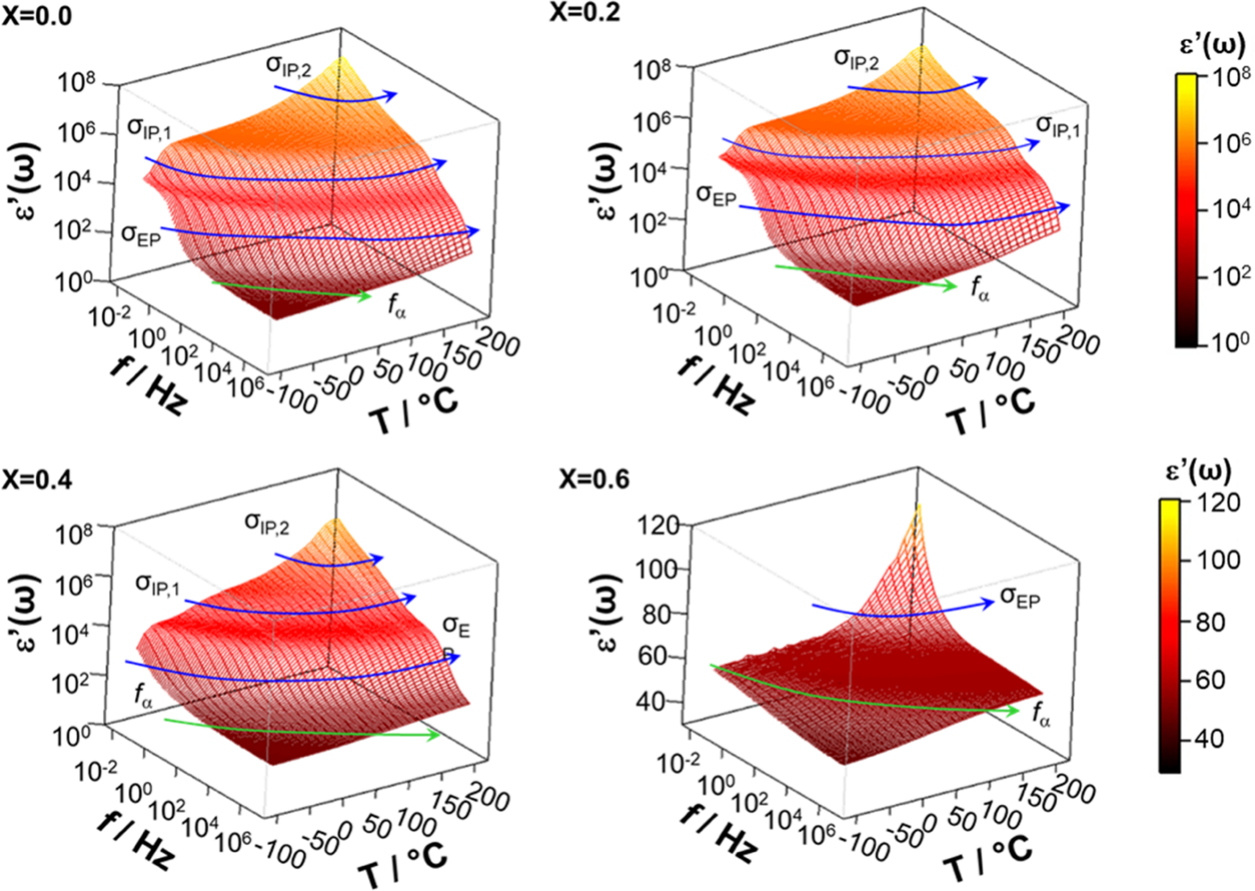
Three-dimensional surfaces of the real component of complex permittivity of four selected perovskite samples with *x* ranging from 0 to 0.6 at various temperatures (−100 ≤ *T* ≤ 150 °C) over the frequency range 30 mHz ≤ *f* ≤ 10^7^ Hz.

**Figure 6:**
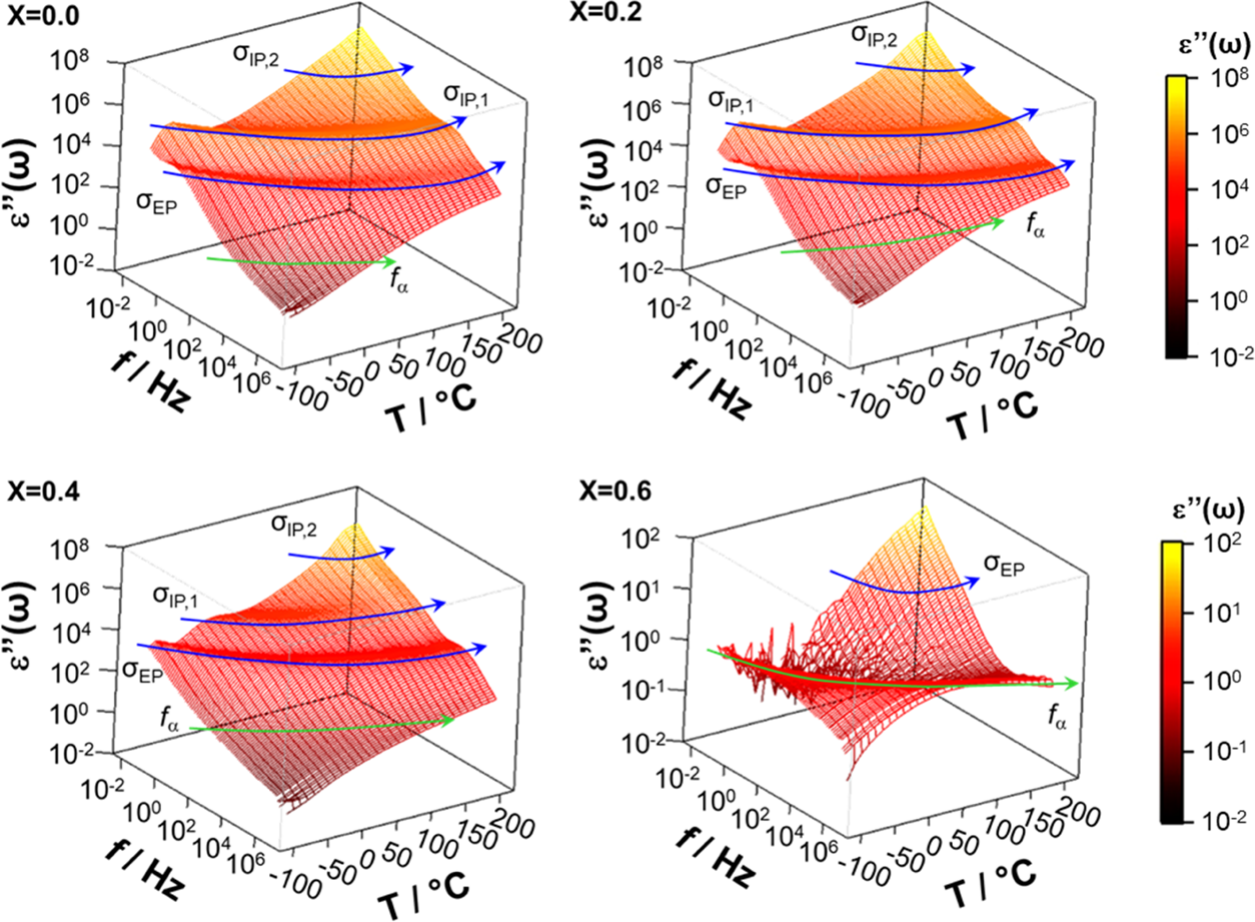
Three-dimensional surfaces of the imaginary component of complex permittivity of four selected perovskite samples with *x* ranging from 0 to 0.6 at various temperatures (−100 ≤ *T* ≤ 150 °C). Measurements were performed in the frequency range 30 mHz ≤ *f* ≤ 10^7^ Hz.

Broadband electric spectroscopy (BES) is a powerful technique to study the electric response of perovskites in terms of dielectric and polarization phenomena. It allows one to reveal at the nanoscale the possible heterogeneities of materials, such as nanodomains and their interfaces. With respect to the Fourier transform infrared (FTIR) and Raman analyses, BES spectroscopy is a dynamic technique that allows one to study the relaxations associated with the host perovskite matrix and with the guest migrating lithium ions. In this way, a complete picture of these events and of their coupling effects is determined. To achieve this target, the electric response of samples is investigated in terms of complex conductivity and permittivity spectra with *x* ranging from 0 to 0.6, in the −100 ≤ *T* ≤ 150 °C temperature range, and in the 30 mHz ≤ *f* ≤ 10^7^ Hz frequency range. This study is crucial to understand the role played by the polarization and dielectric relaxation events on the electric response of the investigated materials (*i.e*., macroscopic conductivities, dielectric strengths, and relaxation times).^38^Figures 5 and 6 show the 3D surfaces of the real (ε′(ω)) and imaginary (ε″(ω)) components of complex permittivity, respectively, for selected perovskites with *x* ranging from 0 to 0.6. Measurements were carried out at various temperatures over the entire frequency range investigated.

The ε′(ω) profiles (Figure 5) present two very intense plateaus followed by a dispersion at high frequency. Corresponding to these latter events, ε″(ω) shows (a) an intense peak that shifts to higher frequencies with temperature and (b) one weak dielectric relaxation event at high frequency. The assignment of these phenomena is corroborated by analyzing the real (σ′(ω)) and imaginary (σ″(ω)) components of complex conductivity (σ*(ω) = σ′(ω) + *i*σ″(ω)) (Figures S3 and S4), which on ln(ω) shows, as expected,^38^ (a) for polarization events, plateaus in σ′(ω) profiles and peaks in σ″(ω) spectra; and (b) for dielectric relaxations, linear steps rising on ω in both σ′(ω) and σ″(ω). On this basis, it is shown that the samples here studied exhibit (Figure 6) three polarization events (*i.e*., the electrode polarization, σ_EP_, and the interdomain polarizations, σ_IP,1_ and σ_IP,2_), and one dielectric relaxation mode (*f*_α_).

**Figure 7:**
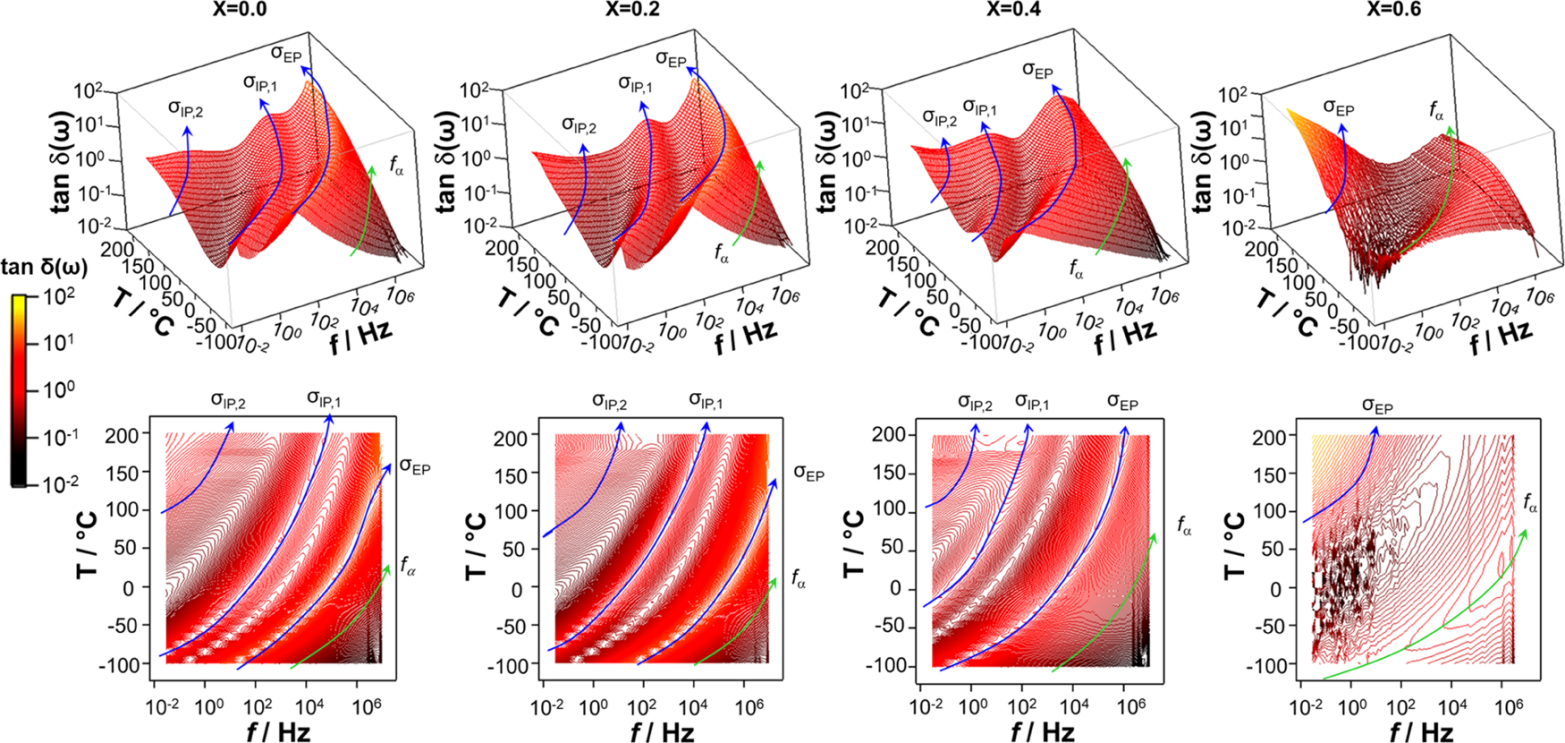
Three-dimensional surfaces (above) and contour plot (below) of tan  δ(ω) for four selected perovskite samples with *x* ranging from 0 to 0.6. The measurements are in the temperature and frequency range −100 ≤ *T* ≤ 150 °C and 30 mHz ≤ *f* ≤ 10^7^ Hz, respectively.

In agreement with other studies,^39^ polarization events show high values of the real component of permittivity. Particularly, σ_EP_ and σ_IP,1_ present ε′(ω) values on the order of 10^3^ relative units. A careful analysis of Figures 5 and 6 reveals that these polarization events on temperature shift to high frequencies, and their ω peak position and intensity are significantly dependent on *x*. Actually, the permittivity of the polarization events, which exceed 10^4^ relative units, decreases on *x*. This demonstrates that the perovskite composition plays a crucial role in modulating the structure and the electric response of materials in this series, which are mainly dominated by σ_EP_ and σ_IP,1_ polarization phenomena. The above assignment is also confirmed by inspecting the 3D surface and contour maps of tan δ(ω) = ε′(ω)/ε″(ω) (see Figure 7), which allow one to identify clearly three polarization events and a weak dielectric relaxation band in the high-frequency wing of spectra at low temperatures. As described elsewhere,^38^ the study of the dependence of the frequency of intense polarization events on the sample thickness allows one to distinguish the electrode polarization (σ_EP_) mode from the two interdomain polarization (σ_IP,1_ and σ_IP,2_) modes. Therefore, the plateau at high frequency corresponds to the conductivity associated with the electrode polarization charge migration pathway (σ_EP_), while those at low frequencies are attributed to σ_IP,1_ and σ_IP,2_ interdomain polarization conductivity pathways (Figure 7). These latter are indicative of the presence in materials at the mesoscale level of structural and/or morphological heterogeneities.

To evaluate the conductivity and the relaxation times associated with the polarization phenomena and the dielectric strength and relaxation time associated with the revealed dielectric relaxation mode, the electric response of materials is analyzed simultaneously in terms of ε*(ω), σ*(ω), and tan δ(ω) spectra by eq 1(38,40,41)
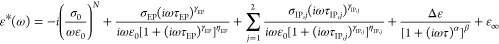
1This is performed considering that  and . The first term of ε*(ω) (eq 1) describes the conductivity of the material at zero frequency (σ_0_), which lies below the frequency of the interval measured experimentally, while ε_∞_ is the permittivity of the material at infinite frequency. The second and third terms account for the electrode and interdomain polarizations, respectively. σ_EP_, σ_IP,j_, τ_EP_, and τ_IP,j_ are the conductivities and relaxation times associated with these latter phenomena. The fourth term expresses the dielectric relaxation, with τ and Δε being the relaxation time and the dielectric strength, respectively, of the *f*_α_ mode (see Figure 7). α and β are its shape parameters that account for the symmetric and asymmetric broadening of the peak. In eq 1, ω = 2π*f* (τ = 1/2π*f*, with *f* in Hz) is the angular frequency of the electric field. A good fit of the data is obtained only when a simultaneous high-quality simulation of ε′(ω), ε″(ω), σ′(ω), σ″(ω), and tan δ(ω) is achieved.^38^

**Figure 8:**
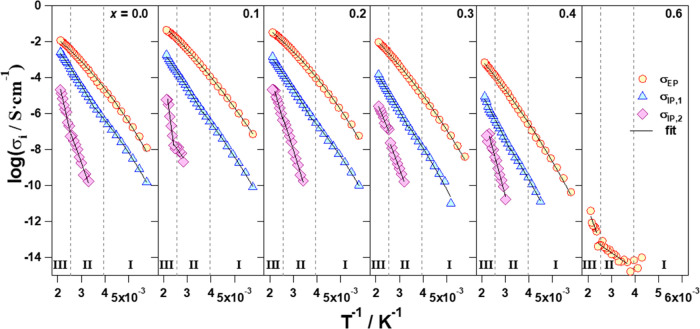
Conductivity of the polarization phenomena (σ_EP_, σ_IP,1_, and σ_IP,2_) as a function of *T*^–1^ for the perovskites at different compositions (0 ≤ *x* ≤ 0.6). Three temperature regions are detected (I, II, III), which are delimited at *T* ≅ −15 and 127 °C.

The values of σ_EP_ and σ_IP,j_ (with *j* = 1 and 2), obtained for samples at different *x*, are plotted on the reciprocal of temperature in Figure 8. The temperature dependence of log σ*_i_* (*i* = EP, IP,1, and IP,2) shows three different temperature regions: I, below *ca*. −15 °C; II, from *ca*. −15 to *ca*. 127 °C; and III, above 127 °C. Except for the material with *x* = 0.6, where only σ_EP_ is detected, in the other samples, the overall conductivity of materials is the superposition of σ_EP_, σ_IP,1_, and σ_IP,2_ (σ_tot_ = σ_EP_ + ∑_*j*=1_^2^σ_IP,j_, with σ_EP_ > σ_IP,1_ > σ_IP,2_). In particular, in the contribution to σ_tot_, σ_EP_ is 2 orders of magnitude higher than σ_IP,1_. This latter is 1 order of magnitude higher than σ_IP,2_, which is of course negligible. Therefore, in the electric response of materials, the overall conductivity is mostly dominated by σ_EP_ (σ_tot_ ≈ σ_EP_).

For *x* = 0, σ_tot_ ≈ σ_EP_ + σ_IP,1_, with σ_IP,1_ significantly contributing to the overall conductivity of this perovskite. On the other hand, when *x* = 0.6, the overall conductivity of the perovskite is coincident with σ_EP_ and shows a value that is at least 6 orders of magnitude lower than that of samples with *x* < 0.6 in the same conditions. This steep conductivity decrease is attributed to a significant reduction of the charge carrier concentration (Li^+^) and to the blockade of the perovskite conduction pathways by La ions (reduction of the A-site vacancies) according to a 3D percolative process.^42^ This inhibits the long-range charge migration phenomena responsible for the high overall ionic conductivity characterizing the other samples. Figure 8 shows that in I, II, and III regions σ_EP_ and σ_IP,j_ (*j* = 1, 2) increase on *T* following a nonlinear behavior. These pieces of evidence demonstrate that in the explored temperature range, the overall conductivity of the proposed materials is the result of the superposition of three ionic conductivity pathways (σ_EP_, σ_IP,1_, and σ_IP,2_). This is in apparent contradiction with other studies^43,44^ on analogous perovskites, which report that only two conductivity pathways are responsible for the overall conductivity of materials, attributed to the ionic and electronic conductivity pathways. Furthermore, on *x*, it is observed that the conductivities of σ_EP_, σ_IP,1_, and σ_IP,2_ decreased gradually and tended to merge together as the concentration of Al rises and Li diminishes (see Figure 8). Finally, a dielectric relaxation (*f*_α_) is revealed in the high-frequency wing of the spectra of complex conductivity and permittivity components. Corresponding to this phenomenon, the profiles of (a) the ε′(ω) and ε″(ω) components show a dispersion and a weak peak, respectively, and (b) the σ″(ω) present a linear behavior. This mode is attributed to the long-range diffusion of BO_6_ (B = Ti, Al) reorientational (LRDR) relaxations in the 3D structure of perovskites, which is coupled with the ionic-exchange events between adjacent vacant sites. This interpretation allows one to reconcile results of studies reported elsewhere for similar systems.^45−48^ On this basis, BES results described here allow us, for the first time, to hypothesize that to maintain fixed the center of mass of the 3D structure of perovskites, the structural fluctuations of BO_6_ octahedra require to be intra- and intercell coupled. In this way, a concerted dynamic event occurs (*i.e*., a segmental modelike phenomenon), which is responsible for the long-range diffusion of lithium cations along the vacant sites of the 2D/3D conductivity pathways of materials.

**Figure 9:**
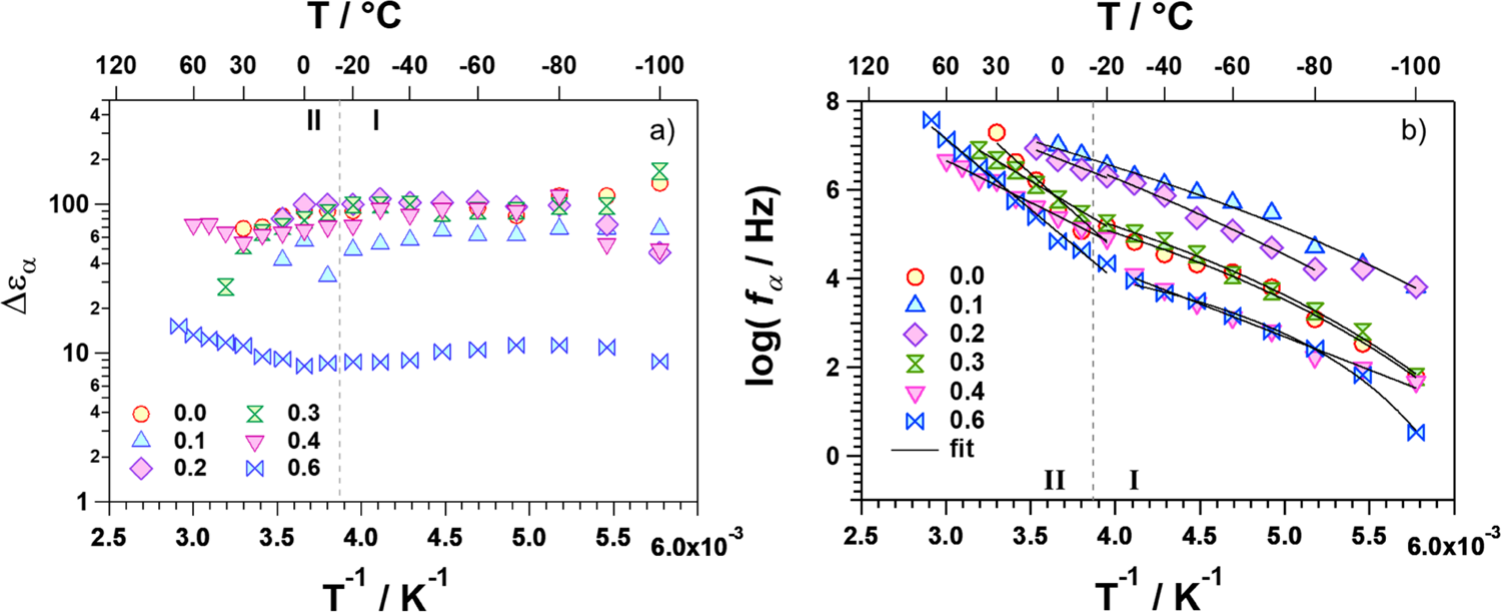
Dependence on *T*^–1^ of the dielectric strength, Δε_α_, (a) and frequency, *f*_α_, (b) of the α relaxation mode for the perovskite samples (0 ≤ *x* ≤ 0.6). I and II temperature regions are delimited at *T* ≅ −15 °C.

The dependence on temperature of the strength (Δε_α_) and of the relaxation frequency (*f*_α_) of the dielectric mode is shown in Figure 9a,b, respectively. Δε_α_ is correlated to the square of the dipole moment per unit volume of material nanodomains.^38^ Its value is correlated (a) to the dipole intensity of fluctuating motions of distorted BO_6_ octahedra (tilting of octahedra) and (b) to their ability to give rise to intra- and intercell dipole–dipole coupling effects within the 3D structure of nanodomains. Δε_α_ values are quite constant on *T*, and in materials with *x* < 0.6, they are higher by at least 1 order of magnitude with respect to the sample with *x* = 0.6 (Figure 9a). This shows that when in the B-site, (a) the Al^3+^ cation is predominating (*x* > 0.4), and the 3D structure of materials is reorganized, reducing the average distortion of BO_6_ octahedra, thus decreasing significantly the average dipole moment per unit volume of material and their 3D coupling effect of dynamic fluctuations; (b) *x* ≤ 0.4, Al^3+^ dispersed in the host matrix acts as a defect, thus facilitating the BO_6_ octahedra fluctuations, which promote the host medium relaxation phenomena of the 3D material domains described above. In this latter case, it is expected that a high density of octahedral distortions acts, rising significantly the dielectric strength of the relaxation mode. To complete these studies, the dependence on temperature of α-relaxation mode frequency (*f*_α_) is investigated (Figure 9b), which, in accordance with Δε_α_*vsT*^–1^ results (Figure 9a), confirms the presence of two temperature regions (I and II) delimited by *T* ∼ −15 °C. Region I presents a Vogel–Tamman–Fulcher (VTF) dependence of *f*_α_ on *T*, confirming that the dynamic distortions of the octahedral B sites act to modulate the long-range flexibility of the 3D material structure, *i.e*., its LRDR mode (Figure 9b). Results show that when Al^3+^ is in the range 0.1 ≤ *x* ≤ 0.2, the LRDR mode of the 3D structure is revealed at high frequencies. Therefore, the long-range flexibility of the 3D network of the perovskite structure is promoted only when Al^3+^ is doping the material in BO_6_ octahedra at a low concentration.

Differently from conventional ion-conducting materials,^4,5,49−52^ the frequency of the α relaxation mode shows a VTF behavior in the low-temperature region (*i.e*., region I, below −15 °C) and a pure linear Arrhenius trend at higher temperatures (*i.e*., region II, *T* > −15 °C). This evidence demonstrates that at low temperatures the coupling effect of structural fluctuation modes of BO_6_ octahedra along the 3D host structure of perovskite domains yields a long-range diffusion of distortional modes. At low temperatures, these long-range host perovskite relaxation modes are coupled with lithium-ion charge migration events. At *T* > −15 °C, a decoupling effect between 3D structural perovskite modes and lithium migration phenomena is revealed.

**Figure 10:**
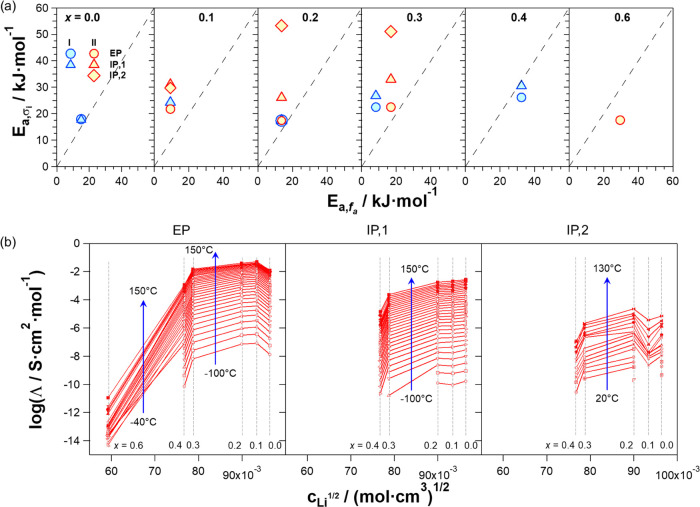
Correlation map between the activation energy values of the polarization phenomena, *E*_*a*,σ_*i*__ (*i* = EP, IP,1, and IP,2), and of the dielectric relaxation modes, *E*_*a,f*_α__, for the perovskites with 0 ≤ *x* ≤ 0.6 (a). Equivalent ionic conductivity (Λ) on c^1/2^ (mol·cm^−3^)^1/2^ (b).

To investigate the influence of the dielectric relaxation mode on the long-range charge migration events characterizing the conductivity pathways of investigated materials, the profiles of *f*_α_ vs *T*^–1^ are simulated as follows: (a) for materials in region II, with *x* > 0.2 and *x* = 0, by Arrhenius-like linear behaviors; and (b) for the other perovskites in region I, by VTF-like behaviors. The map of Figure 10a correlates the pseudo-activation energies of conductivity pathways (*E*_*a*,σ_*i*__) with values determined on *f*_α_ vs *T*^–1^ profiles (*E*_*a,f*_α__). This demonstrates that in I and for samples in II with *x* ranging from 0.1 to 0.2, the pseudo-activation energy of the dielectric mode exhibits values on the same order of magnitude (*ca*. 20 kJ·mol^−1^) as those of σ_EP_ and σ_IP,1_.

This observation highlights that the long-range migration events of lithium ions along the conductivity pathways (σ_EP_, σ_IP,1_, and σ_IP,2_) are directly correlated to the long-range relaxation phenomena of the inorganic host network, which is associated with the long-range intra- and intercell coupling fluctuations of tilting octahedra in different crystallographic domains (*P*4/*mmm* or *R*3̅*c*). Results of Figure 10a,b show that this phenomenon (a) is enhanced when *x* ranges from 0.1 to 0.2 and (b) is significantly inhibited when *x* = 0 and 0.6 (*i.e*., when no Al^3+^ is present in the material or when it is the predominant species into the B sites of the 3D octahedral structure of the perovskite).

Further confirmation of the effect of percolation pathways of vacancies, yielded by Al^3+^ doping ions into the host 3D network, can be revealed by studying the dependence of the equivalent conductivity (Λσ*_i_*/[Li^+^], I = EP, IP1, and IP2) of polarization events on *c*^1/2^ (mol/cm^3^)^1/2^ of Li^+^ (see Figure 10b). Λ values are obtained using σ*_i_* data of Figure 8; the sample stoichiometry and experimental density values are summarized in Table S2. Results show that Λ (a) is higher in the 0 < *x* < 3 region and decreases steeply beyond *x* = 0.3 and (b) presents values decreasing in the order Λ(EP) > Λ(IP1) > Λ(IP2) with reducing values of at least 2 orders of magnitude from EP to IP2 modes.

**Figure 11:**
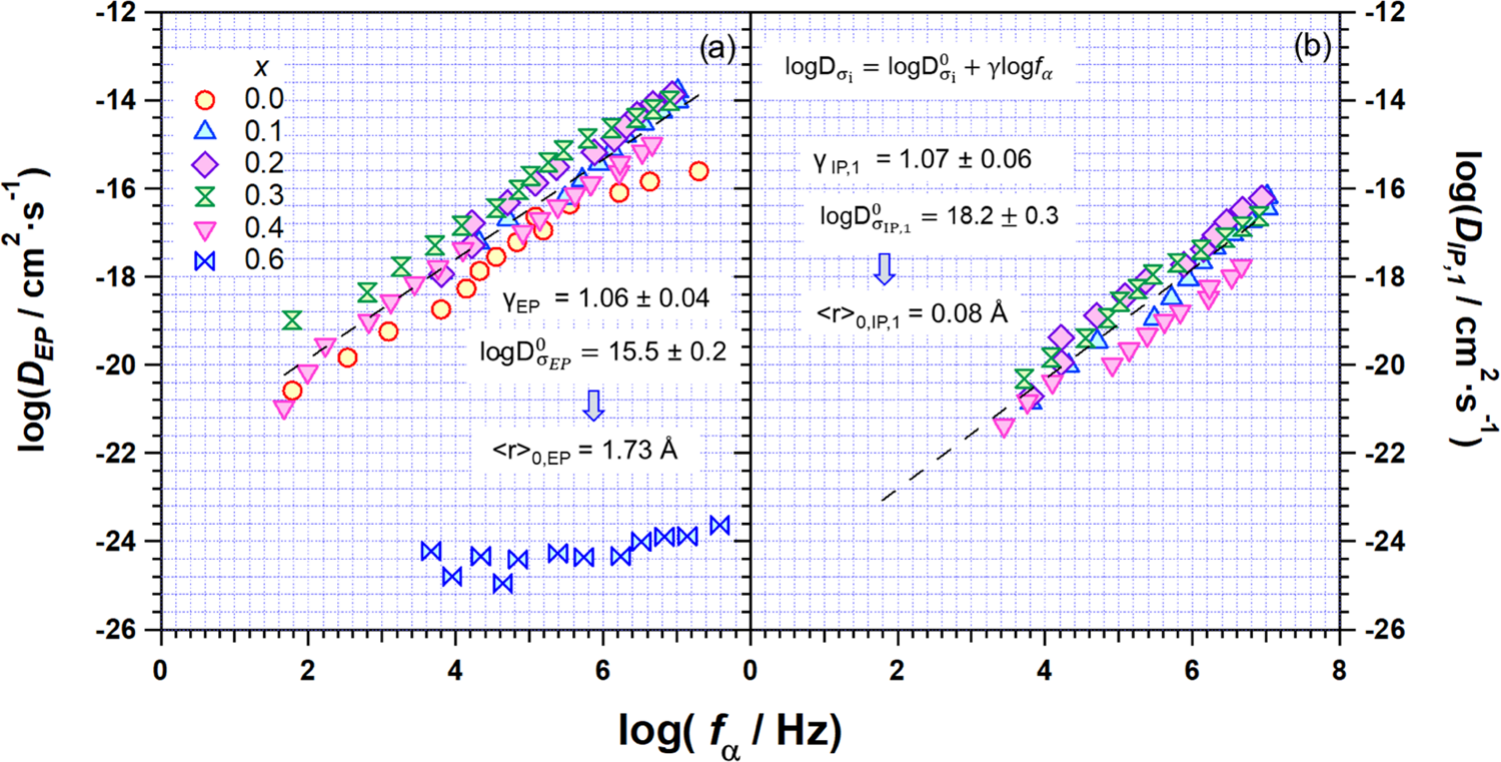
Correlation map between the diffusion coefficients of the polarization phenomena, *D*_σ*_i_*_ (σ*_i_* = σ_EP_ and σ_IP,1_), and the *f*_α_ relaxation mode of perovskites with 0 ≤ *x* ≤ 0.6. *D*_σ_*i*__ are evaluated as described in the text. Dashed lines show the Einstein–Smoluchowski-like fit obtained using eq 3.

To better clarify the influence of the dielectric relaxation on the conductivity mechanism pathways, the diffusion coefficients *D*_EP_ and *D*_IP,1_ of each σ*_i_* are calculated and correlated to the frequency of the α-relaxation mode. To determine *D*_*i*_ values, the Nernst–Einstein relation in the form of eq 2 is used^53^
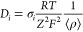
2where *D*_*i*_ and σ*_i_* are the diffusion coefficients and the conductivity values of the EP and IP,1 pathways, respectively. *R* and *F* are the gas and Faraday constants, respectively. *T* is the temperature in K. *Z* and ⟨ρ⟩ are the charge and the average density of the carrier, respectively. ⟨ρ⟩, which is assumed constant for all of the σ*_i_* pathways, is determined considering the experimental values of the density of perovskites summarized in Table S2. Figure 11a,b shows that both *D*_EP_ and *D*_IP,1_ values are well correlated to the *f*_α_ of perovskites. In particular, it is revealed that the effect of the relaxation mode of the 3D host structural network of these materials (*i.e*., the LRDR mode) is more effective in stimulating the EP charge migration pathway rather than that of the IP,1 phenomenon. Actually, at the same *f*_α_, *D*_EP_ exhibits values of at least 2 orders of magnitude higher than that of the IP,1 event. Furthermore, it is to be noted that at *x* > 0.2, *D*_EP_ and *D*_IP,1_ seem to be less stimulated by the *f*_α_ relaxation mode. This shows that a large concentration of vicariant Al^3+^ in the BO_6_ octahedral framework acts to inhibit the coupling phenomena between long-range charge migration events and the host matrix relaxation. The dashed line of Figure 11a,b is obtained by considering for lithium migration a pure 3D Einstein–Smoluchowski percolation behavior.^53,54^ This well reproduces the experimental data when it is in the linear form
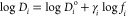
3where  and  with τ_*i*_ being the relaxation time of the *i*-th mode. Fitting parameters of both *D*_EP_ and *D*_IP,1_*vs f*_α_ are shown in Figure 11a,b. It is revealed that (a) when γ_EP_ ≅ γ_IP,1_ ≅ 1, the diffusion coefficients of these modes are perfectly correlated to the perovskite’s LRDR mode involving the long-range coupling of tilting fluctuations of BO_6_ octahedral units; and (b) in conditions when ⟨*r*_0_⟩_EP_ and ⟨*r*_0_⟩_IP,1_ are 1.73 and 0 Å, respectively, no lithium migration between adjacent empty sites is possible along the 3D structure of these materials.

In details, the ⟨*r*_0_⟩_EP_ value suggests that the lithium cation is fluctuating around its coordination site center with a large amplitude, confirming that in the EP pathway the 3D structural relaxation mode of the perovskite domain network is more effective. In this way, in EP pathways at high temperatures, the mobility of lithium ions is facilitated owing to the coupling effect between the relaxation events of host medium relaxations (LRDR motion) and the migration processes between dynamic coordination sites of Li^+^ ions (vacancies).

**Figure 12:**
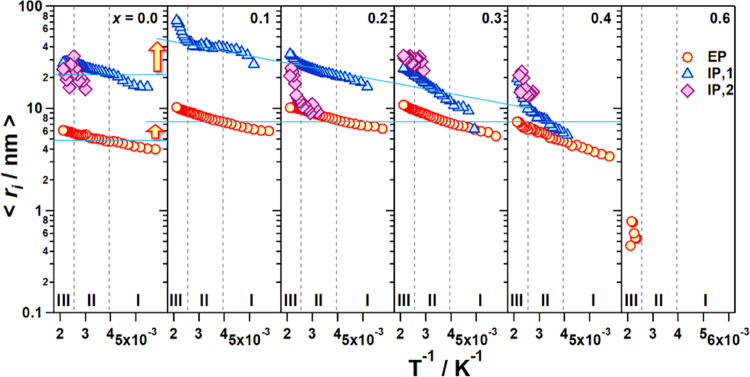
Dependence of the average Li^+^ migration distances, ⟨*r*_i_⟩ (with *i* = EP, IP,1, and IP,2), on *T*^–1^ for the perovskite samples with 0 ≤ *x* ≤ 0.6. ⟨*r*_i_⟩ values are determined using eq 4 (see text).

Furthermore, insights into the conductivity mechanisms of perovskites are obtained by analyzing the effect on temperature of the average lithium migration distance, ⟨*r*_*i*_⟩ (see Figure 12), calculated from the following equation^55,56^
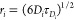
4where *D*_*i*_ and τ_*D*_*i*__ are the diffusion coefficient and the relaxation time of the *i*-th polarization event, respectively. In accordance with other studies,^57,58^ ⟨*r*_*i*_⟩ increases as the temperature rises for all of the investigated materials. Figure 12 shows the following.

(1) At *x* = 0.6, the long-range charge migration phenomena are irrelevant for EP. This demonstrates that when in B sites Al^3+^ ions exceed the Ti^4+^ concentration (a poor Li^+^ content with a conduction pathway blockade by La ions, *i.e*., a negligible concentration of A-site vacancies), the structural relaxation modes are highly inhibited and the coupling of these latter phenomena with migration events of Li^+^ ion is negligible.
(2) At *x* = 0, only Ti^4+^ ions are present; thus, the Li^+^ content is maximum and the tilting fluctuation of BO_6_ octahedra is facilitated. A significant splitting of almost 1 order of magnitude between the average migration distance values of EP and IP pathways is disclosed. A comparison of the average migration distance of this sample with that of the perovskite with *x* = 0.6 shows an increase of ⟨*r*_EP_⟩ of at least 1 order of magnitude.
(3) For *x* = 0.1 and 0.2, the Li^+^ content is relatively high and Al^3+^ ions act as defects stimulating the local fluctuation modes of BO_6_ units (α relaxation). Intra- and intercell coupling of these latter modes with lithium long-range migration events is responsible for the significant rise in these samples, with respect to *x* = 0, of the ⟨*r*_EP_⟩ and ⟨*r*_IP_⟩ values with ⟨*r*_IP_⟩ > ⟨*r*_EP_⟩.
(4) At *x* > 0.2, the diminishing Li^+^ content accounts for the decrease in both ⟨*r*_EP_⟩ and ⟨*r*_IP_⟩ on *T*^–1^ as well as for the trend to merge together, reducing their splitting. This confirms that a large content of Al^3+^ in B sites reduces the tilting relaxations of BO_6_ octahedra and their coupling with lithium migration phenomena. Therefore, a decrease in *x* of the long-range migration distances of both types of Li^+^ conductivity pathways is observed.

Taken all together, EP and IP conductivity pathways for Li^+^ long-range migration phenomena are very effective when in these perovskites, (a) a sufficient density of charge carriers (Li ions and A-site vacancies) is present and (b) in the Ti^4+^-based BO_6_ backbone octahedra, Al^3+^ ions act as defects, thus enhancing the dynamics characterizing their relaxation modes. This latter effect influences the coupling phenomena between relaxations of the inorganic 3D host network and the relaxation events characterizing the long-range migration processes of lithium conductivity pathways.

### DFT and Molecular Simulation

**Figure 13:**
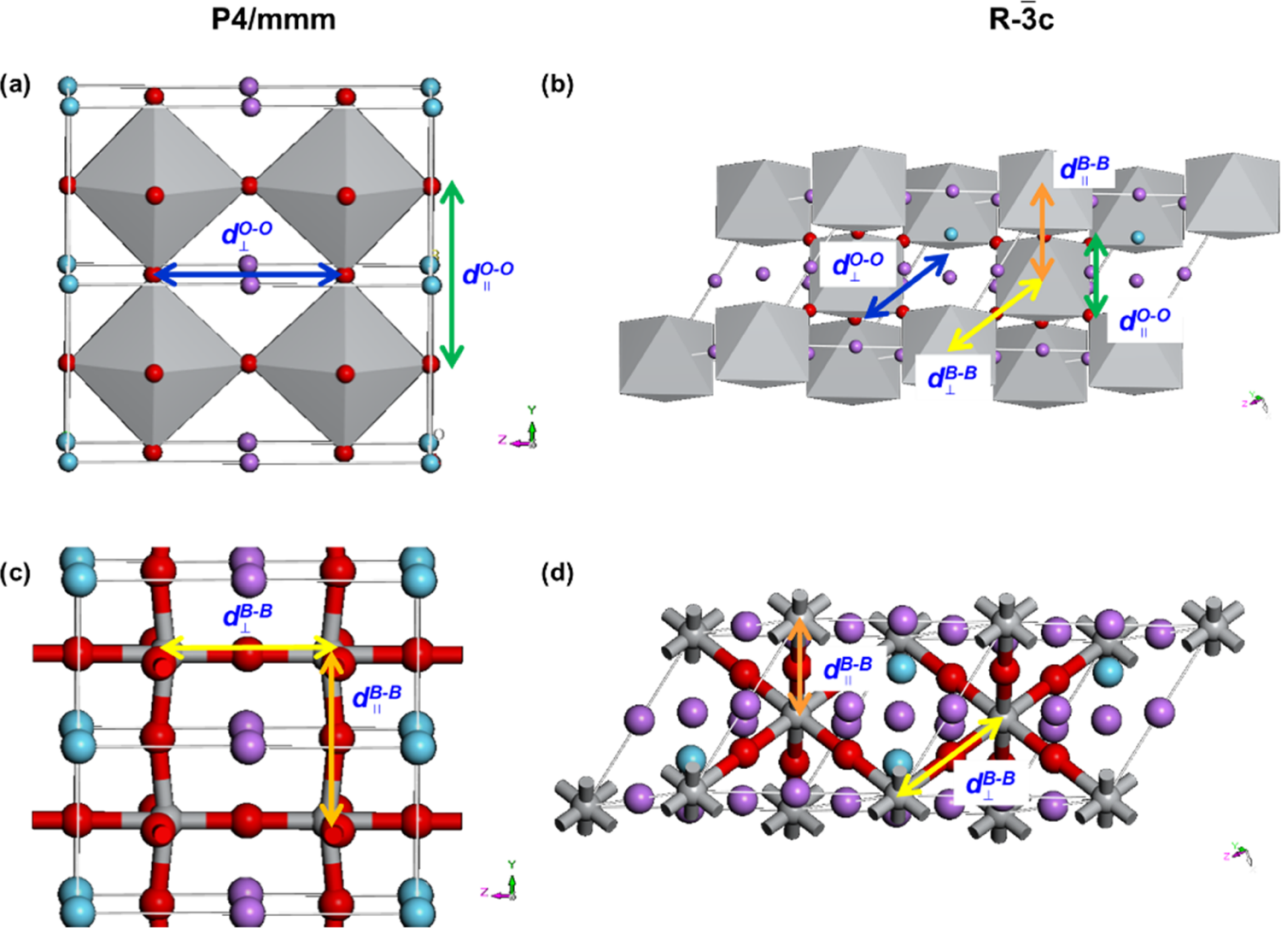
Schematic representation of a selected La_0.6_Li_0.4_Ti_0.8_Al_0.2_O_3_ (*x* = 0.2) perovskite with crystal structures *P*4/*mmm* (a, c) (*a* = *b* = 4.035601 Å, *c* = 8,09171 Å; α = β = γ = 90°) and *R*3̅*c* (b, d) (*a* = *b* = *c* = 5,479742 Å; α = β = γ = 60.01156°) (red ring solid = O; purple ring solid = Li; blue ring solid = La, black ring solid =Ti, Al). Oxygen–oxygen and B–B (B = Ti, Al) atomic distances are labeled as follows: for *P*4/*mmm*, *d*_00*l*_^O–O^ ≡ *d*_∥_^O–O^(*P*), *d*_0*k*0_^O–O^ ≡ *d*_⊥_^O–O^(*P*), *d*_0*k*0_^B–B^ ≡ *d*_⊥_^B–B^(*P*), *d*_00*l*_^B–B^ ≡ *d*_∥_^B–B^(*P*); and for *R*3̅*c*, *d*_*hkl*_^O–O^ ≡ *d*_∥_^O–O^(*R*), *d*_*h*0*l*_^O–O^ ≡ *d*_⊥_^O–O^(*R*), *d*_0*kl*_^B–B^ ≡ *d*_∥_^B–B^(*R*), and *d*_*hk*0_^B–B^ ≡ *d*_⊥_^B–B^(*R*).

To clarify in more details at an atomic level the phenomena responsible for the conductivity pathways of the investigated materials, DFT calculations and dynamic molecular simulation were performed, as described in the Experimental Section. For each composition in the series, the structure was first investigated minimizing its energy by DFT calculations. The obtained results were then used as starting points to study the overall dynamics of the systems by means of dynamic molecular simulations, which allowed one to shed light on (a) the long-range Li^+^ migration events and (b) the structural relaxations of the inorganic networks. These latter phenomena are responsible for the experimental conductivity pathways revealed by BES. Based on the results obtained in the microstructural characterization described above, the starting point of the simulation was to reproduce by DFT calculations the *P*4/*mmm* and *R*3̅*c* experimentally observed crystal structures (see Figure 13) of materials. The Ti/Al and La/Li atomic ratios corresponding to the nominal compositions of materials were considered for the atomic occupancy. For the sake of completeness, the above simulations were also carried out in both of the above-considered space groups by the sitting Li^+^ cation, as suggested elsewhere,^17^ out of the A-site in the position (0.45, 0, 0). Results thus obtained are labeled *P*4/*mmm*(Ls) and *R*3̅*c*(Ls).

Simulations were performed by adopting a box as described in the Experimental Section at 25 °C for 10 ps. The average energy and the structural modulations are measured every 1 fs. To better understand the complex structural dynamics of perovskites, the two symmetry space groups described above are adopted and the oxygen–oxygen (O–O) and B–B distances (B = Ti, Al) shown in Figure 13 are measured *vs* simulation time. For the *P*4/*mmm* (*P*) domains of materials, the following distances are determined: (a) *d*_00*l*_^O–O^ ≡ *d*_∥_^O–O^(*P*) and *d*_0*k*0_^O–O^ ≡ *d*_⊥_^O–O^(*P*), which are indicative of the bottleneck in lithium-ion elementary migration events and of the tilting distortions of BO_6_ octahedra (Figure 13a); and (b) *d*_0*k*0_^B–B^ ≡ *d*_⊥_^B–B^(*P*) and *d*_00*l*_^B–B^ ≡ *d*_∥_^B–B^(*P*), which on the other hand probe the possible network distortions among the centers of the BO_6_ octahedra. For domains in the materials with the *R*3̅*c* space group (Figure 13b,d), the following average distances are measured: (a) *d*_*hkl*_^O–O^ ≡ *d*_∥_^O–O^(*R*) and *d*_*h*0*l*_^O–O^ ≡ *d*_⊥_^O–O^(*R*) to test the dynamics of oxygens of BO_6_ octahedra; and (b) *d*_0*kl*_^B–B^ ≡ *d*_∥_^B–B^(*R*) and *d*_*hk*0_^B–B^ ≡ *d*_⊥_^B–B^(*R*) for evaluating the network distortions and the cooperative interactions characterizing fluctuating BO_6_ octahedra. The analysis of the behavior on *x* of the above ⊥ to // distances of materials in the two different space groups is crucial for revealing the possible presence of the cooperative dynamic pseudo-Jahn–Teller phenomena, which are likely responsible for the host medium relaxation phenomena detected by BES spectroscopy. In this context, long-range diffusion of BO_6_ octahedra distortional modes, which take place owing to intercell dipole–dipole interactions, is possible only when the following conditions are both fulfilled: (a) the values of *d*_*j*_^α^ (*j* = ||, ⊥) should be lower than 4 Å and (b) *d*_⊥_^O–O^ ≈ *d*_∥_^B–B^ in the BO_6_ octahedral repeat units of the cells of material domains.

**Figure 14:**
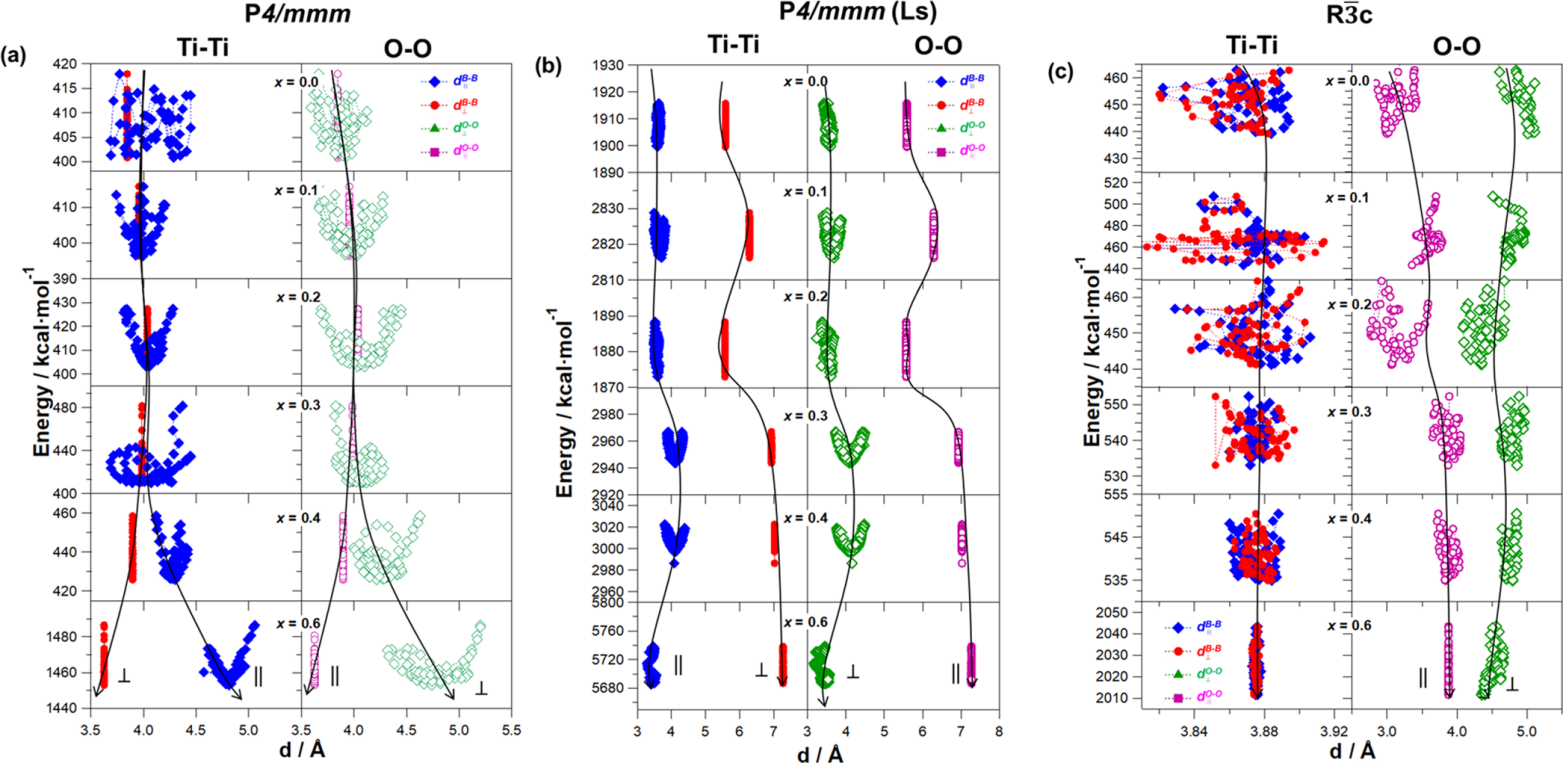
Dependence of the overall energy on *d*_*J*_^α^ (α = (O–O), (B–B); *J* = ⊥ //) for perovskites simulated in the space groups (a) *P*4/*mmm*, (b) *P*4/*mmm*(Ls), and (c) *R*3̅*c*. *P*4/*mmm*(Ls) is the *P*4/*mmm* space group with Li^+^ into the (0,0,0.05) position.

Figure 14 shows the dependence of the overall energy of the systems at different *x* on the *d*_*J*_^O–O^ and *d*_*j*_^B–B^ distances. The following are to be observed for *P* materials.

(a) With *P*4/*mmm* and *x* ranging from 0 to 0.3 and with *P*4/*mmm*(Ls) and at all *x*, it results that *d*_*j*_^α^ ≤ 4 Å and *d*_⊥_^O–O^(*P*) = *d*_∥_^B–B^(*P*). This behavior shows that the dynamic pseudo-Jahn–Teller distortions of sites A and B octahedra are totally symmetric. Therefore, in the 3D network of the material, these relaxation modes are perfectly coupled with each other, facilitating the Li^+^ long-range migration events. It should be observed that the d fluctuations present a minimum of energy around *d* ≅ 3.9 Å. This value is in agreement with the criterion proposed above (*d*_*j*_^α^ ≤ 4 Å), which is consistent with the diameter of the bottleneck for lithium-ion conduction in perovskites with similar compositions (3.871 Å).^45,48,59^ In these latter, it is reported that O^2–^ ions in the TiO_6_ octahedra exhibit an ionic radius of 1.40 Å,^17,45^ thus allowing one to determine that the bottleneck for lithium conduction is 1.07 Å. This value is only *ca*. 3% lower than that revealed here for the investigated materials (1.10 Å). Taken all together, at *x* ≤ 0.3 for *P*4/*mmm* and at all values of *x* for *P*4/*mmm*(Ls), the dynamic pseudo-Jahn–Teller effects (*d*_⊥_^O–O^(*P*) = *d*_∥_^B–B^(*P*)) are cooperative and coupled in an intra- and intercell way, thus (a) enhancing the long-range structural relaxation phenomena (LRDR motions) of perovskites and (b) facilitating the long-range migration events of the Li^+^ cation. Obviously, this is the only effective way for obtaining an efficient 3D coupling in host inorganic matrices between charge migration and host medium relaxation events.
(b) With *P*4/*mmm* at *x* > 0.3, it is obtained that *d*_∥_^B–B^(*P*) ≅ *d*_⊥_^O–O^(*P*) while *d*_*j*_^α^ > 4 Å. Here, the long-range coupling of the antisymmetric dynamic pseudo-Jahn–Teller phenomena is inhibited by symmetry, thus giving rise to confined BO_6_ rotational motions in the cell of the host matrix. These are distortional modes, which are likely decoupled from each other and from the Li^+^ long-range charge migration events, thus decreasing the overall conductivity of materials.

For perovskites in the *R*3̅*c* space group, the dependence on *x* of *d*_*j*_^B–B^ and *d*_*j*_^O–O^, with *j* = || or ⊥, is shown in Figure 14c. The ⟨*d*_*j*_^B–B^⟩ and ⟨*d*_∥_^O–O^⟩ present a value of *ca*. 3.87 Å, which is in perfect agreement with other similar perovskite materials.^17,45^ However, *d*_⊥_^O–O^, which is higher than ca. 4.50 Å, is decoupled from *d*_∥_^B–B^, thus probing that no coupling in the long-range distance of dynamic pseudo-Jahn–Teller modes occurs when the perovskites are in the *R*3̅*c* space group. In this condition, no effect of the LRDR mode of the 3D host matrix on the Li^+^ long-range migration phenomena is expected, which results in a decrease in the conductivity of the different pathways of this phase.

**Figure 15:**
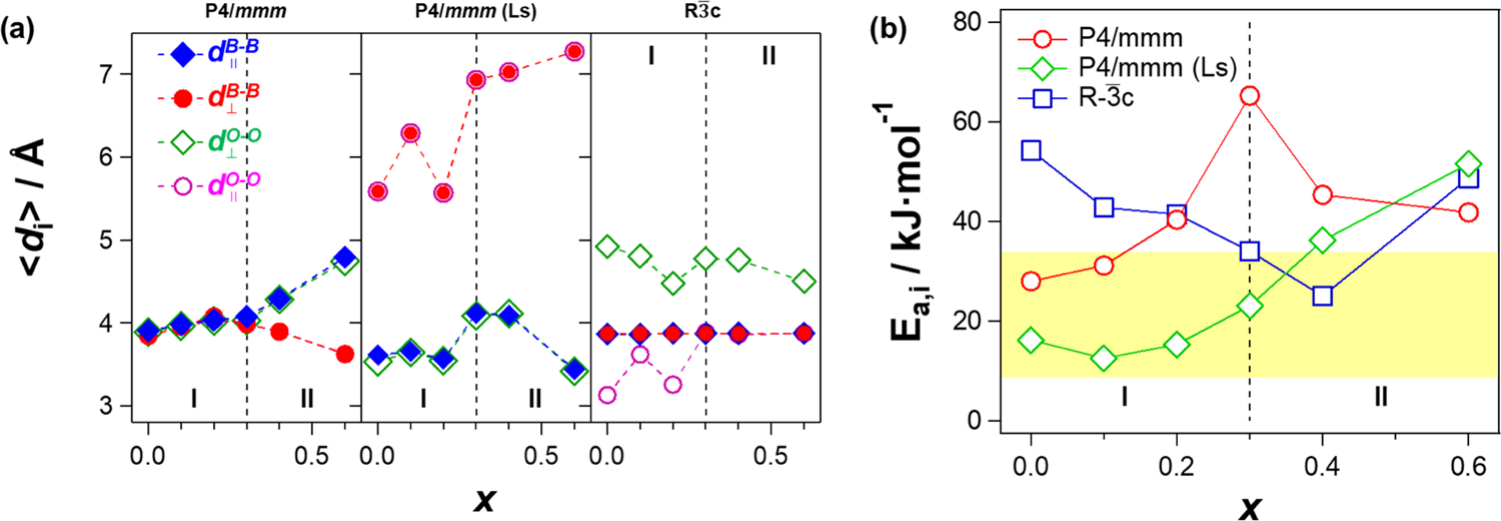
Dependence on *x* of ⟨*d*_*j*_^α^⟩ (a) and *E*_α,β_ (the simulated activation energy) (b). Simulations are performed for perovskites in *P*4/*mmm* and *R*3̅*c* space groups. The yellow region highlights the range of activation energy values determined by σ_EP_ and σ_IP,1_ profiles shown in Figure 12.

The dependence on *x* of the average distances, ⟨*d*_*j*_^α^⟩, with α = Ti–Ti, O–O, and *j* = ⊥ //, clearly confirms these behaviors (Figure 15a) and reveals that, in the two investigated space groups, the 3D network relaxation modes of the materials act to define two *x* regions (I and II). In I, for *P*4/*mmm* domains at *x* ≤ 0.3 and in all *x* range of *P*4/*mmm*(Ls), the two simulated host relaxation modes (*d*_⊥_^O–O^(*P*) = *d*_∥_^B–B^(*P*)) are completely coupled, while they are totally decoupled for domains with the *R*3̅*c* space group. In II, at *x* > 0.3, simulated structural relaxations present a significant decoupling between ⊥ and || modes. This increases on *x* for the *P*4/*mmm* space group and decreases for *R*3̅*c*.

Figure 15b shows the dependence on *x* of the simulated average activation energy, *E*_σ,β_, for the materials with β = *P*4/*mmm*, *P*4/*mmm*(Ls), and *R*3̅*c*. *E*_σ,β_ is the average activation energy determined by evaluating the difference between the value obtained by averaging the energy at each fluctuating *d* value (Figure 14) and the minimum energy detected at a given *x*. Results show that, in the two energy regions, the behavior on *x* of the average activation energy (Figure 15b) (a) is on the same order of magnitude of values measured by BES studies; (b) in I, for materials in *P*4/*mmm* and *P*4/*mmm*(Ls), it is lower than that of *R*3̅*c* domains and coinciding with *E*_*a,*σ_*i*__ and *E*_*a,f*_α__ (Figure 10); and (c) in II, with respect to *E*_*a,*σ_*i*__ and *E*_*a,f*_α__, for *R*3̅*c* materials at *x* < 0.6, it is on the same order of magnitude, while it is higher for both *P*4/*mmm* and *P*4/*mmm*(Ls). This emphasizes that, for an efficient long-range charge migration phenomenon, the coupling of LRDR motions of the host network with the long-range Li^+^ migration events is crucial. In the systems here investigated, the LRDR mode is modulated by the long-range coupling of dynamic pseudo-Jahn–Teller distortions of the repeat units of host material domains. A comprehensive movie showing the Li^+^ migration pathways in investigated perovskites (*i.e*., *P*4/*mmm, P*4/*mmm*(Ls), and *R*3̅*c*(Ls)) at *x* = 0 is reported in the Web Enhanced Objects 1–3. In these movies, it can be observed how the complex host relaxation modes of the perovskite networks, with a particular reference to the fluctuations of BO_6_ octahedra, are coupled with the lithium-ion migration events.

## Conclusions

Crystals of the different compositions in the La_1/2+1/2*x*_Li_1/2–1/2*x*_Ti_1–*x*_Al*_x_*O_3_ series of fast ionic conductors display a very complex nanostructure constituted by a mixture of two different ordered nanoregions of tetragonal *P*4/*mmm* and rhombohedral *R*3̅*c* symmetries, as revealed by combining XRD and HRTEM/STEM. For samples with a low Al content, the conductivity values are on the order of *ca*. 10^–3^ S·cm^–1^. These values drop down with Al concentration by several orders of magnitude owing to the blockage of the conductivity pathways along the three-dimensional network of materials, which is caused by the La incorporation and the vacant A-site reduction. Broadband electric spectroscopy measurements have allowed a detailed analysis of the relaxation modes to investigate the “dynamic” properties of the structural network and reveal the influence of the nanostructural features on the long-range diffusion of lithium cations along the two network conductivity pathways (*i.e*., σ_EP_ and σ_IP_). Experimental observations have been rationalized at the atomic level using density functional theory (DFT) calculations and dynamic molecular simulation. BES results here reported allow us to reveal, for the first time, that the structural tilting fluctuations of BO_6_ octahedra are able to give rise to intra- and intercell coupling phenomena. In this way, a concerted 3D network dynamic event can be formed (similar to segmental movements of polymeric chains), which, when coupled with elementary migration events of Li^+^, is responsible for the long-range diffusion of lithium cations along the vacant sites of the two detected network conductivity pathways. Correlation of dynamic molecular simulation studies with BES results allows one to conclude that in the investigated perovskites

(a) both *P*4/*mmm* and *R*3̅*c* domains of materials are involved in the electric response of materials when *x* < 0.6;
(b) the coexistence of these domains is responsible for the interdomain conductivity pathways, σ_IP,i_;
(c) the conductivity associated with the electrode polarization pathway (σ_EP_) is reasonably associated with the superposition of the conductivity contribution due to both the investigated structural domains composing the materials σ_EP_ = σ_EP_(*P*4/*mmm*) + σ_EP_(*P*4/*mmm*(Ls)) + σ_EP_(*R*3̅*c*) with σ_EP_(*P*4/*mmm*(Ls) ≥ σ_EP_(*P*4/*mmm*) ≫ σ_EP_(*R*3̅*c*)); indeed, as *x* rises, the amount of *P*4/*mmm* domains decreases and that associated with the *R*3̅*c* space group increases;
(d) σ_EP_ is directly related to the LRDR motion of material domains in both space groups; in general, this latter phenomenon is more effective for conductivity pathways in *P*4/*mmm* domains at *x* < 0.3; and
(e) σ_IP,1_ is indicative of the presence of nanodomains with different space groups and disappears in the perovskite at *x* = 0.6, when the material can be considered as consisting of a single domain with the *R*3̅*c* space group.

## Supporting Information Available

High-resolution TEM images of crystals corresponding to *x* = 0.2, 0.4, and 0.6 compositions in the [110]_p_ zone axis; representation of the *R*3̅*c* S.G., *P*4/*mmm*, and *R*3̅*c* S.G. cells; 2D profiles of real conductivity as a function of frequency at different temperatures; 2D profiles of imaginary conductivity as a function of frequency at different temperatures; table including structural models used for refinement and calculations of rhombohedral and tetragonal perovskites; table including theoretical and measured densities of materials (PDF)
Movie clips showing the coupling between fluctuating BO_6_ octahedral motions and lithium-ion migration events in the *P*4/*mmm* structure (AVI)
Movie clips showing the coupling between fluctuating BO_6_ octahedral motions and lithium-ion migration events in the *P*4/*mmm*(Ls) structure (AVI)
Movie clips showing the coupling between fluctuating BO_6_ octahedral motions and lithium-ion migration events in the *R*3̅*c*(Ls) structure (AVI)

## Supplementary Material

cm2c00459_si_001.pdf

cm2c00459_si_002.avi

cm2c00459_si_003.avi

cm2c00459_si_004.avi
