# Supplementary material for: Effect of Relaxations on the Conductivity of La1/2+1/2xLi1/2–1/2xTi1–xAlxO3 Fast Ion Conductors
Source: Chem Mater. 2022 Jun 6;34(12):5484–99. doi: 10.1021/acs.chemmater.2c00459 (PMC9245440; doi:10.1021/acs.chemmater.2c00459)
Supplement: Supplementary file 1 — cm2c00459_si_001.pdf [file cm2c00459_si_001.pdf]

Supporting Information

Effect of Relaxations on Conductivity of $La_{1/2+1/2x}Li_{1/2-1/2x}Ti_{1-x}Al_xO_3$ Fast Ion Conductors

Keti Vezzù[§], Ester García-González[⊥], Gioele Pagot[§,†], Esteban Urones-Garrote[#], Maria Eugenia Sotomayor[‡], Alejandro Varez[‡], Vito Di Noto[§,†,*]

[§]Section of Chemistry for the Technology (ChemTech), Department of Industrial Engineering, University of Padova, Via Marzolo 9, I-35131 Padova (PD), Italy

[⊥] Departamento de Química Inorgánica. Facultad de Ciencias Químicas, Universidad Complutense, Madrid 28040, Spain

†Centro Studi di Economia e Tecnica dell'Energia Giorgio Levi Cases, Via Marzolo 9, I-35131 Padova (PD), Italy

[#]Centro Nacional de Microscopia electrónica, Facultad de Ciencias Químicas, Universidad Complutense, Madrid 28040, Spain

[‡]Materials Science and Engineering Department, University Carlos III of Madrid, Av. de la Universidad 30, Leganés, E-28911 Madrid, Spain

*Corresponding author: phone number +39 049 8275229, email vito.dinoto@unipd.it (VDN)

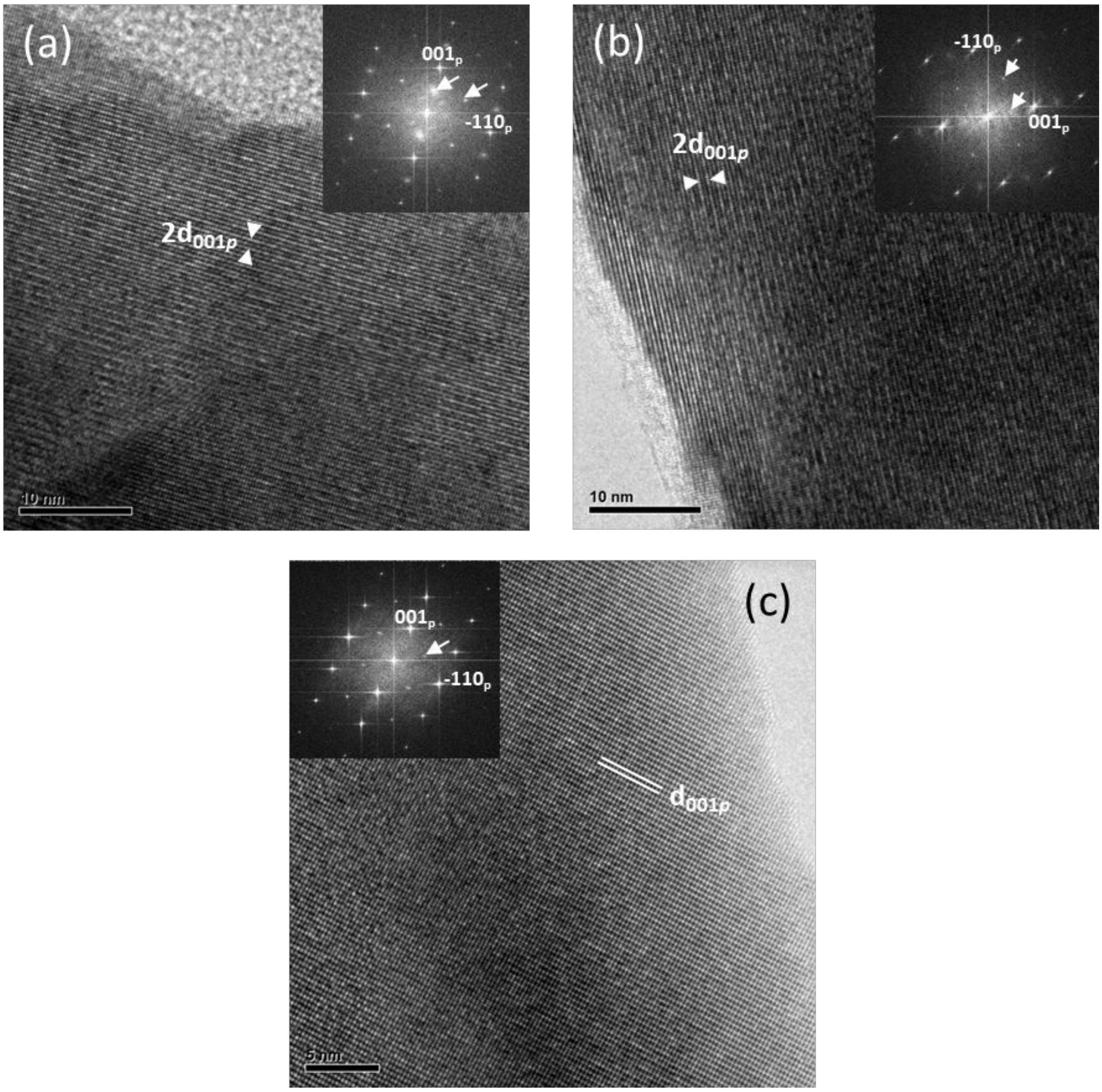

Figure S1. High resolution TEM images of crystals corresponding to x = 0.2 (a), 0.4 (b), and 0.6 (c) compositions in the $[110]_p$ zone axis (*p* subindex refers to the basic perovskite cell). Arrows indicate the extra diffraction maxima with respect to the basic perovskite cell.

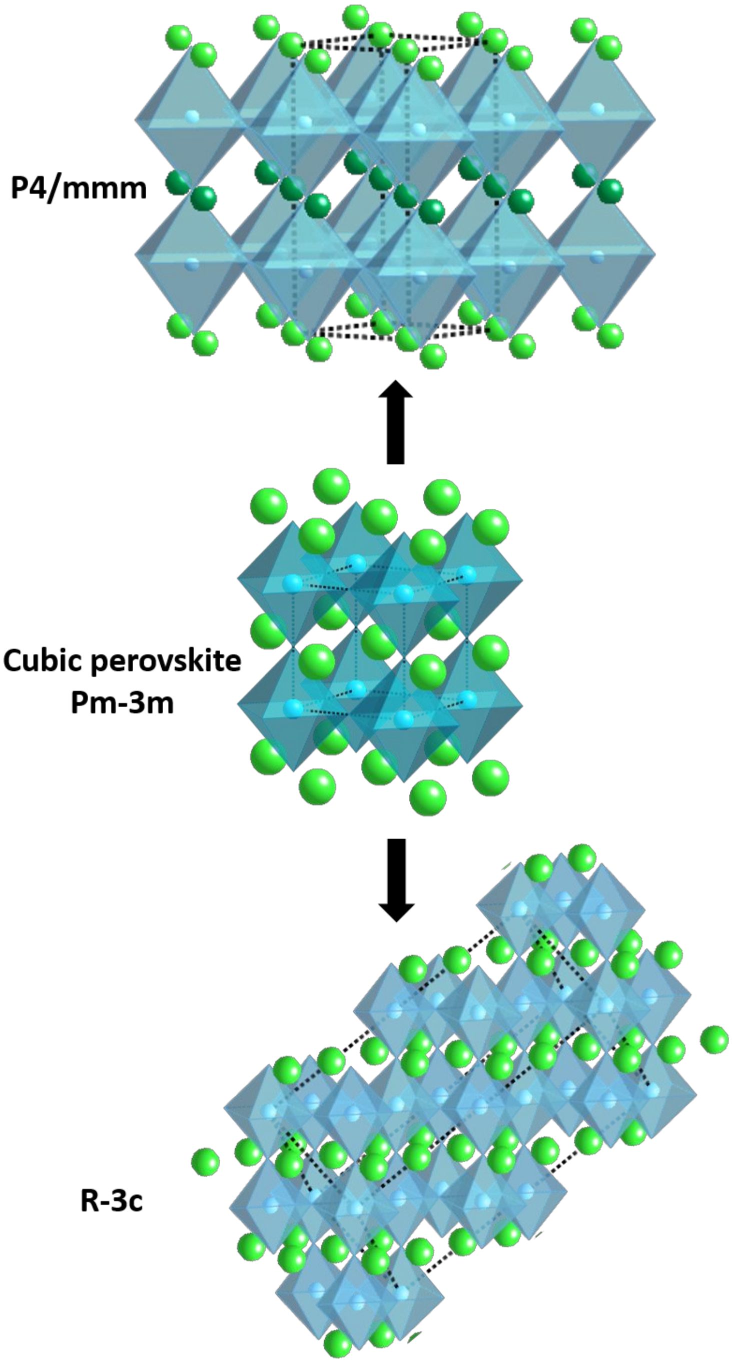

Figure S2. Relationship between the basic perovskite unit cell (P*m-3m* S.G.) and the two cells of the structural domains found in the crystals (P*4/mmm* and R-*3c* S.G.). Blue polyhedra correspond to Ti(Al) atoms and green balls represent La atoms (note there are two different positions for lanthanum in the tetragonal P4/mmm cell, as mentioned in the text).

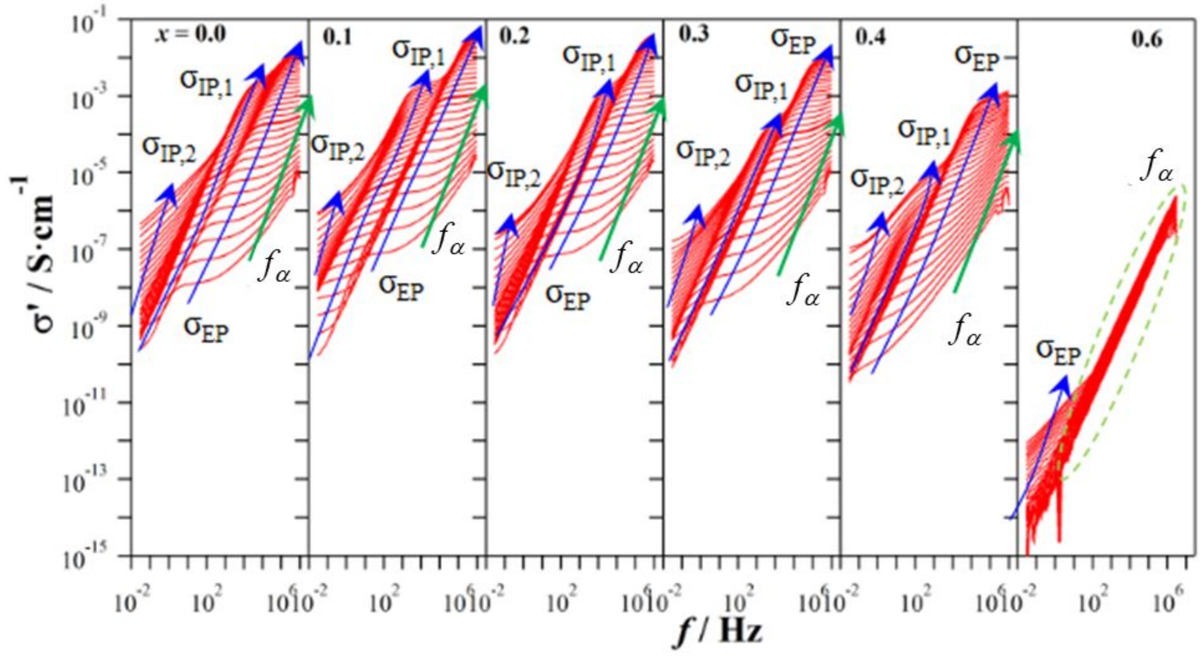

Figure S3. 2D profiles of real conductivity as function of f (30 mHz $\leq f \leq 10^7$ Hz) and T (-100 - 150°C) for the six samples of perovskite ($0 \leq x \leq 0.6$).

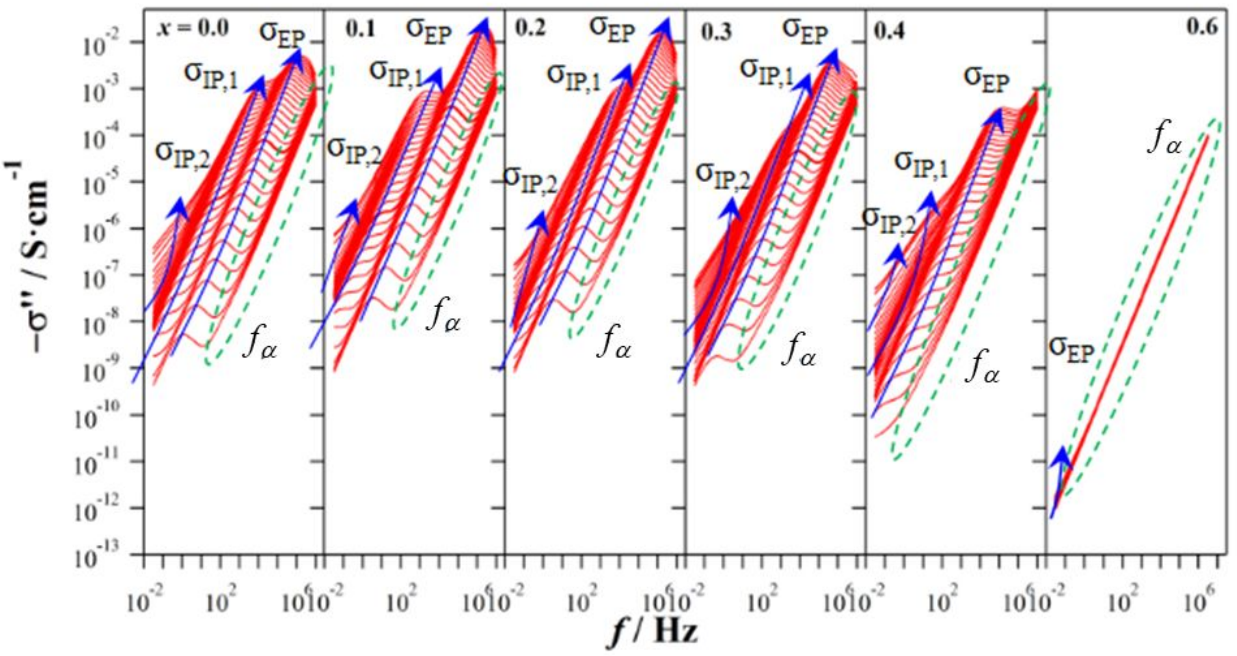

Figure S4. 2D profiles of imaginary conductivity as function of f (30 mHz $\leq f \leq 10^7$ Hz) and T (-100 - 150°C) for the six samples of perovskite ($0 \leq x \leq 0.6$).

Table S1. Structural models used for refinement and calculations of rhombohedral and tetragonal perovskites.

		S.G. R-3c[a)]			S.G P4/mmm[b)]
Atom	Position	Atomic coordinates	Atom	Position	Atomic coordinates
La,	*6a*	0, 0, ¼	La1	*1a*	0, 0, 0
Ti	*6b*	0, 0, 0	La2	*1b*	0, 0, ½
O	*18e*	x ≈½ , 0, ¼	Ti	*2h*	½, ½, z
			O1	*1c*	½, ½, 0
			O2	*1d*	½, ½, ½
			O3	*4i*	0, ½, z

a) Taken from reference Alonso, J. A.; Sanz, J.; Santamaría, J.; León, C.; Várez, A.; Fernández-Díaz, M. T., On the Location of Li^+ Cations in the Fast Li-Cation Conductor $La_{0.5}Li_{0.5}TiO_3$ Perovskite. *Angewandte Chemie International Edition* **2000,** *39* (3), 619-621.

b) Taken from reference Ibarra, J.; Várez, A.; León, C.; Santamaría, J.; Torres-Martínez, L. M.; Sanz, J., Influence of composition on the structure and conductivity of the fast ionic conductors $La_{2/3-x}Li_{3x}TiO_3$ ($0.03 \leq x \leq 0.167$). *Solid State Ionics* **2000,** *134* (3-4), 219-228.

Table S2. Theoretical and measured densities of materials with nominal composition $La_{1/2+1/2x}Li_{1/2-1/2x}Ti_{1-x}Al_xO_3$ (x = 0.2, 0.4 and 0.6)

	x = 0.2	x = 0.4	x = 0.6
Theoretical Density (g·cm^{-3})	5.0930	5.4477	5.7762
Measured Density (g·cm^{-3})[a] (true density)	5.015(6)	5.379(7)	5.693(8)

[a] Measured by means of a Helium Pycnometer.
